# Effects of Dietary-SCFA on Microbial Protein Synthesis and Urinal Urea-N Excretion Are Related to Microbiota Diversity in Rumen

**DOI:** 10.3389/fphys.2019.01079

**Published:** 2019-08-22

**Authors:** Zhongyan Lu, Hong Shen, Zanming Shen

**Affiliations:** ^1^Key Laboratory of Animal Physiology and Biochemistry, College of Veterinary Medicine, Nanjing Agricultural University, Nanjing, China; ^2^College of Life Sciences, Nanjing Agricultural University, Nanjing, China; ^3^Bioinformatics Center, Nanjing Agricultural University, Nanjing, China

**Keywords:** diet, short chain fatty acid, urea transporter B, microbial protein, urinal urea-N, rumen microbiota

## Abstract

Two experiments were performed in this study. In Experiment 1, twenty goats were fed with an isonitrogenous diet, containing 28% Non-Fiber Carbohydrate (MNFC group, *n* = 10) or 14% NFC (LNFC group, *n* = 10). In the MNFC group, the ruminal concentration of Short Chain Fatty Acids (SCFA) increased, and pH declined. Compared with those in the LNFC group, the microbial protein synthesis in rumen and mRNA abundance of urea transporter B (UT-B) in rumen epithelium increased in the MNFC group, although serum urea-N (SUN) did not differ significantly between groups. Simultaneously, urinal urea-N excretion was reduced in the MNFC group. Significant correlations were found between rumen SCFA and UT-B and between UT-B and urinal urea-N excretion. Furthermore, the abundances of SCFA receptor of GPR41 and GPR43 increased in the rumen epithelium of the MNFC group. These results suggest that increases of SUN transported into the rumen and incorporated into microbial protein and decreases of urinal urea-N excretion are related to ruminal SCFA. This is supported by data from our previous study in which added SCFA on the mucosal side caused increases of urea transport rate (flux J_sm_ urea) from the blood to the ruminal lumen side. In Experiment 2, we used 16S rRNA Amplicon Sequencing to analyze the structure of the ruminal microbiota community in relation to SCFA. An additional eight goats were assigned into the MNFC (*n* = 4) and LNFC (*n* = 4) groups. The dietary ingredients, chemical composition, and feeding regimes were the same as those in Experiment 1. Constrained correspondence analysis (CCA analysis) revealed NFC promoted the expansion of microbiota diversity, particularly of SCFA-producing microbes. The function prediction of 19 upregulated Kyoto Encyclopedia of Genes and Genomes (KEGG) ortholog groups showed an NFC-induced increase of the types and abundances of genes coding for enzymes catalyzing N and fatty acid metabolism. Based on our present and previous investigations, our results indicate that, in goats consuming a MNFC diet, the facilitated urea transport in the rumen and improved urea N salvage are triggered by an expansion of ruminal microbiota diversity and are signaled by ruminal SCFA. This study thus provides new insights into the microbiota involved in the dietary modulation of urea-N salvage in ruminant animals.

## Introduction

Urea is one of the nitrogen resources involved in ruminant protein metabolism. Urea is produced in the liver via the ornithine-urea cycle and is passed into the blood through the hepatic vein. Liver-synthesized urea can be either recycled back to the digestive tracts or excreted into urine (Stewart and Smith, 2005; Reynolds and Kristensen, 2008). In the digestive tract of ruminants, the rumen is the largest and most important compartment for nutrient digestion and absorption. In the rumen, microbes capture the ammonia (NH_3_) released from urea (Houpt and Houpt, 1968) and incorporate it into protein that enables microbial growth (Kiran and Mutsvangwa, 2010). The rumen microbes, mixed with digesta, flow into the hind gut. In the small intestine, the microbial protein releases amino acids and nucleic acids through enzymolysis (Fujihara and Shem, 2011). In this way, some of the urea-N, derived from the recycling pathway, is converted into host-available N and is deposited in the host body (nitrogen salvage). However, the excretion of N in urine results not only in the loss of an N resource, but also influences water quality, e.g., coastal eutrophication (Glibert et al., 2006). The dominant form of N in the urine of ruminant animals is urea (Marini and Van Amburgh, 2003). In the urine of young Holstein cattle fed on a silage-based diet, the accumulated N excretion amounts to 30.6 kg/year/animal (Jiao et al., 2014). Urea can be rapidly hydrolyzed upon excretion and is subsequently transformed into nitrogen-containing substances classified as pollution: NH_3_, NO_3_^–^, or N_2_O (Dijkstra et al., 2013). These substances produce nitrogen-containing fine particulate matter in air and lead to the formation of ground-level ozone, greenhouse gas, and other forms of reactive nitrogen (Sutton and Bleeker, 2013). Hence, a reduction in urinal urea-N is urgently required for sustainable ruminant husbandry and environmental protection.

The recycling of urea to the rumen accounts for 40–80% of liver synthesized urea (Harmeyer and Martens, 1980). This rate varies greatly with diet (Stewart and Smith, 2005), although the mechanism underlying this variation is not fully understood. The fractions of urea entry transferred to the gut or excreted in the urine are related in a reciprocal manner (Reynolds and Kristensen, 2008). Accordingly, the more that urea enters the rumen, the less that it is secreted into the urine, highlighting the crucial role of the blood urea transport in the processes of N utilization and urinal urea-N excretion reduction. In ruminant animals, the transfer of urea from the blood to the rumen occurs via direct passage through the rumen wall and via saliva secretion. A previous study (Houpt, 1959) has shown that the total urea-N transfer for sheep is 5.2 mM urea-N/h, of which a transfer rate of 0.3 mM/h has been measured in saliva, demonstrating the important role of the former route in urea recycling. Similar results have been observed in the goat. Numerous studies have revealed that major plasma urea entry to the rumen is mediated by the urea transporter (Stewart, 2011). The urea transporter UT-B is known to be expressed in the ovine and bovine rumen epithelium and to facilitate blood urea transport across the rumen epithelium (Stewart and Smith, 2005; Lu et al., 2014). Enhancement of the ruminal urea flux (Lu et al., 2014) and microbial protein production are recognized as being dependent on the dietary carbohydrate source and on rumen microbial fermentation (Hoover and Stokes, 1991) Our previous studies have shown the increasing abundance of UT-B mRNA and protein, stimulated by microbial metabolites, i.e., short chain fatty acids (SCFA) and acidic pH, in goats ingesting easily fermentable carbohydrate (Lu et al., 2015). Moreover, SCFA and acidic pH have been found to enhance urea flux across the rumen epithelium in *ex vivo* studies (Abdoun et al., 2010; Lu et al., 2015). SCFA are the most abundant microbial metabolites in the rumen. In recent years, growing interest has been shown in the interaction between gut microbiota and host for health and disease (Clemente et al., 2012). Diet represents a link between the microbiota and host physiology in human and mono-stomach animals (Koh et al., 2016) and in ruminant animals (Hook et al., 2011). In the rumen, easily fermentable carbohydrate, e.g., non-fiber carbohydrate (NFC), expands microbiota diversity (Shen et al., 2016). Ruminal NFC promotes rumen fermentation and SCFA production (Aschenbach et al., 2011). However, excessive NFC diets fed to ruminant animal exert adverse effects. In a previous study, Hook et al. (2011) have reported that a high proportion of starch (409 g kg^–1^ DM) fed to cows causes a decline of rumen pH to 5.6, a change in bacteria community structure represented by a decline of cellulolytic bacteria and an increase of lactate producer, a rise in lactate production, and subsequently subacute ruminal acidosis. This occurs because fibrolytic ruminal bacteria are sensitive to low pH (Asanuma and Hino, 1997). The growth of the main types of cellulolytic bacteria is inhibited below pH 5.9 (Russell and Dombrowski, 1980), but lactic-acid-producing bacteria can still function under these conditions (Asanuma and Hino, 1997). Nevertheless, whether the rumen microbiota, which are the producers of SCFA, are involved in the dietary regulation of N utilization is not fully understood. We have hypothesized that a MNFC diet (containing 28% Non-Fiber Carbohydrate) causes the expansion of ruminal microbiota diversity and SCFA concentration, with SCFA inducing UT-B expression and microbial protein synthesis, leading to a reduction of urinal urea-N excretion.

To understand the effect of NFC on urinal urea-N excretion, twenty eight goats were used in two experiments. In Experiment 1, twenty goats were fed isonitrogenously on a hay-based diet supplemented with two different percentages of NFC. In order to obtain insights into the effects of dietary NFC-SCFA on urinal urea-N excretion, we measured the ruminal SCFA concentration, UT-B expression, serum urea-N concentration, and urinal urea-N excretion. This was followed by an analysis of the correlation between SCFA and UT-B expression and between UT-B expression and urinal urea-N excretion. In addition, the role of SCFA on UT-B expression was confirmed by the determination of SCFA receptors GPR41 and GPR43 in rumen epithelium. To improve our understanding of the role of SCFA on N deposition in the goat body, N incorporation into the rumen microbial protein was measured. Experiment 2 was designed to elucidate the relationship between ruminal SCFA and microbiota diversity. An additional eight goats were studied by 16S rRNA gene sequencing in order to determine rumen microbiota diversity. We also analyzed genus diversity of SCFA producer and its correlation to SCFA concentration.

## Materials and Methods

### Ethics Statement

This study was approved by the Animal Care and Use Committee of Nanjing Agricultural University, in compliance with the Regulations for the Administration of Affairs Concerning Experimental Animals (No. 588 Document of the State Council of China, 2011).

### Animal Experiments

#### Experiment 1

Twenty goats (Boer × Yangtze River Delta White, 4-month-old, 16.8 ± 1.2 kg BW), housed in individual pens, were randomly allocated into two groups and received an isonitrogenous diet (Supplementary Table S1) of either 600 g of dried hay plus 260 g of concentrate (MNFC, containing NFC: 28% DM, *n* = 10) or 770 g of dried hay plus 90 g of concentrate (LNFC, containing NFC: 14% of DM, *n* = 10). Goats in both groups were fed two equal portions of the designed diet at 0800 and 1700 h daily for 28 day. During the course of the experiment, the dietary energy level [700 kJ/(kg^0.75^⋅day)] was ∼1.4 × maintenance in MNFC and [600 kJ/(kg^0.75^⋅day)] ∼1.2 × maintenance in LNFC group. Fresh water was freely available to all goats during the feeding trial. Urine was collected, over a 24 h period (Huntington, 1989), from day 22 to day 23 of the feeding trial (for urine collection, see the next paragraph: “Collection of urine”) and body weight was measured. On day 29, 10 out of 20 goats (5 from each group) were slaughtered to collect the rumen content and epithelium at a local slaughterhouse at 6 h after the morning feeding. On day 30, the remaining 10 goats were slaughtered by the same method. Immediately after slaughter the rumen content and rumen epithelium were sampled and stored for the determination of rumen parameter and epithelial gene expression (For details please see the paragraph of “Collection of ruminal and blood sample” and “Laboratory analysis”). Dietary intake was measured and recorded daily for each goat throughout the feeding trial, and the feed stuff were adjusted to ensure the desired ratio of hay to concentrate and 30% refuse. The chemical analysis of the feed stuff was performed during week 1 and the last week of the feeding trial (Supplementary Table S1).

#### Experiment 2

An additional eight goats (Boer × Yangtze River Delta White goats, 4-month-old, 17.3 ± 1.1 kg of BW) were randomly allocated into two groups and were fed with an isonitrogenic diet containing 28% NFC (MNFC, *n* = 4) or 14% NFC (LNFC, *n* = 4). The dietary ingredients, chemical composition, and feeding regimes were the same as those in Experiment 1.

### Collection of Urine

During the urea collection period, goats from both groups were kept in individual metabolic cages. A funnel-shaped rubber urine collector (diameter: 15 cm) was strapped to the goat in order to prevent fecal contamination of the urine samples. This collector lay 0.5–1 cm directly below the abdomen of the goat and subsequently passed urine into a collecting vial through a rubber tube by gravity. Sufficient 10% H_2_SO_4_ was added to the vial to maintain a pH ≤ 3. The total amount of collected urine over a 24 h period (Huntington, 1989; Wickersham et al., 2008) was used to measure urinal urea-N excretion. Immediately after collection, the urine volume was measured, and the pH was determined with a pH meter (Mettler Toledo Delta 320; Mettler Toledo Group, Halstead, United Kingdom). The samples were then transferred to −20°C until further analysis.

### Collection of Ruminal and Blood Sample

In experiment 1, after the stunning of the animal by a captive bolt and exsanguination at a local slaughterhouse, the reticulorumen together with the omasum was removed from the abdominal cavity within 2–3 min. The rumen was opened, and the whole content was collected and mixed. Subsequently, 50 ml rumen content was collected from each goat and strained through four layers of cheese cloth. The pH was measured immediately by a pH meter (Mettler Toledo Delta 320; Mettler Toledo Group, Halstead, United Kingdom). Samples of 30 mL filtrated rumen fluid transferred into plastic bottles containing 5% HgCl_2_ solution to inactivate microbes and then stored at −20°C until analysis of rumen SCFA concentration, pH, NH_3_-N, and microbial protein. Rumen tissue from the ventral blind sac (1 cm × 1 cm) was excised, rinsed, placed in RNAlater solution overnight and then stored at −80°C until analyzed for mRNA for UT-B, GPR41, and GPR43, as described below. Blood samples were collected by jugular venipuncture into 5-mL EDTA-coated Vacutainer tubes on the last day of Experiment 1, at 1 h before morning feeding. The blood samples were immediately placed on ice, centrifuged at 1500 *g* at 4°C for 15 min to obtain serum, and then stored at −20°C until further analysis. In Experiment 2, the processes of slaughter and the obtaining of the rumen content were the same as those in Experiment 1. After the rumen content had been strained through four layers of cheese cloth, the pH was measured immediately, as in experiment 1. Samples of 20 mL filtrated rumen fluid were collected from each goat as described in Experiment 1 and stored at −20°C for biochemical analysis. An additional 15 ml filtrated rumen fluid was collected and stored at −20°C for rumen microbiota analysis.

### Laboratory Analysis

Ruminal SCFA concentrations were measured using a GC HP6890N (Agilent Technologies, Santa Clara, CA, United States), as described previously (Lu et al., 2015) In brief, the GC HP6890N was equipped with a flame ionization detector and a 30 m× 0.32 mm internal diameter × 0.25 μm film thickness HP-FFAP capillary column (Hewlett-Packard, Palo Alto, CA, United States). The carrier gas was N_2_ (99.99% purity) adjusted to a constant flow rate of 2.8 ml/min and a split ratio of 1:30. The temperature of the capillary column was set to 140°C for 4 min and was then raised by 25°C/min to 240°C. The temperatures of the injection port and the flame ionization detector were 180 and 250°C, respectively. Crotonic acid was used as an internal standard. In this paper, the term “SCFA concentration” refers to the sum of acetate, propionate, and butyrate concentrations. The rumen-liquid-associated MCP concentration was measured by a BCA Protein Assay Kit, following the instruction of the supplier (Nanjing Jiancheng Taihao Biological Technology Co., Ltd., Nanjing, China). Before measurement, the rumen fluid was pretreated as follows. In brief, the rumen fluid was mixed by a magnetic stirrer at 400 × *g*, 45 s, followed by centrifugation at 408 × *g*, 5 min. Then, 1 ml supernatant was collected and centrifuged at 21000 × *g* for 30 min. The precipitate was resuspended in McDougall’s buffer and centrifuged at 21000 × *g*, for 30 min. McDougall’s buffer is an artificial ovine saliva widely used in rumen microbial culture (McDougall, 1948; Knowles et al., 2017). This process was repeated three times. Subsequently, 2 ml of a 0.25 M NaOH solution was added to the precipitate, which was bathed in boiling water for 10 min and then centrifuged at 21000 × *g* for 30 min. An aliquot of 20 μl of supernatant was taken, and the liquid-associated microbial protein concentration was measured by the BCA method. Serum urea-N was determined by the diacetylmonoxime method. The biochemical reagents and rumen samples were prepared as previously reported (Wybenga et al., 1971). A UV-2100 spectrophotometer (Unico Instruments Co., Ltd., Shanghai, China) was used at a wavelength of 540 nm to determine the urea-N concentration. A phenol-hypochlorite assay was used to measure the NH_3_-N concentration, the biochemical reagents, rumen samples, and NH_4_Cl standards being prepared as described by Broderick and Kang (1980). The absorbance of rumen samples and standards was determined at a wavelength of 630 nm with a UV-2100 spectrophotometer.

Microbial N synthesis was directly estimated from urinary purine derivatives (PD). The urine excreted PD (PDe) was calculated as the sum of allantoin and uric acid in urine (Chen et al., 1990; Kozloski et al., 2017). The concentration of allantoin was measured with a commercial ELISA kit (MyBioSource, United States), and uric acid was determined by using a commercial kit (Sigma-Aldrich, St. Louis, MO, United States). The protocols of the assay were according to the manufacturer’s recommendations.

Microbial N synthesis was calculated as follows:

(1) Microbial⁢N⁢(g/d)=(PDa× 70)/(0.116× 0.83× 1,000)= 0.727×PDa

The amount of PDa (microbial purines absorbed) corresponding to the PDe was calculated as:

PDa (mM/day) = 0.84 × PDe + (0.15 × BW^0.75^ e (^−0.25×*PD*)^) (Chen et al., 1990; Soltan et al., 2018).

Total RNA was isolated from ruminal tissue from the ventral blind sac of goats in Experiment 1 (for sampling and storage, see previous section) by using the RNeasy Mini Kit (Qiagen, Shanghai, China). Isolated mRNA was quantified by using a NanoDrop 1000 spectrophotometer, and its integrity was evaluated by using the RNA 6000 Assay Kit of the Agilent Bioanalyzer 2100 system (Agilent Technologies, Santa Clara, CA, United States). A random hexamer primer (Invitrogen, Shanghai, China) and moloney murine leukemia virus (M-MLV) reverse transcriptase (Fermentas, Burlington, ON, Canada) were employed to synthesize the cDNA according to the manufacturer’s instructions. Quantitative PCR was performed by using the StepOne Plus real-time PCR system (Applied Biosystems, Den Ijssel, Netherlands) and SYBR-Green (Roche, Shanghai, China) for detection. Real-time PCR was carried out in a total volume of 20 μL containing 1 × iQ SYBR Green supermix (Bio-Rad Laboratories, Inc., Hercules, CA, United States), a mixture of forward and reverse primers (500 nM each), 1 ng cDNA template, and sterile water. An initial cycle of 30 s at 95°C was used to denature the cDNA. This was followed by 45 PCR cycles consisting of denaturation at 95°C for 5 s and primer annealing and extension at 54°C for 30 s for UT-B and at 54°C for 30 s for GPR41 and GPR43. Glyceraldehyde 3-phosphate dehydrogenase (GADPH) was chosen as the housekeeping gene. The primer of the UT-B gene was synthesized according to the description of Kiran and Mutsvangwa (Kiran and Mutsvangwa, 2010). The primers of GAPDH, GPR41, and GPR43 genes were designed according to the available sequences in Genbank (Supplementary Table S2). The amplification efficiency of the primers (between 97.5 and 99.7%) was determined by means of a dilution series of epithelial cDNA. All samples were run in triplicate, and the data were analyzed according to the 2^–ΔΔCT^ method (Livak and Schmittgen, 2001). The identity and purity of the amplified product were checked by analysis of the melting curve obtained at the end of the amplification.

### Ruminal Microbiota Analysis

The total DNA of the microbiota was extracted from the ruminal fluid by using a Bacterial DNA Kit (Omega). The DNA concentration was determined in a Nanodrop 1000 (Thermo Fisher Scientific, Wilmington, DE, United States), and the DNA was stored at −20°C until further processing. The 16S rRNA amplicon library preparation was performed by PCR amplification of the V3–V4 region of the 16S rRNA gene with the universal primers 319F (5′-ACTCCTACGGGAGGCAGCAG-3′) and 806R (5′-GGACTACHVGGGTWTCTAAT-3′) (Mori et al., 2014) All libraries were sequenced on an Illumina MiSeq platform (Illumina, San Diego, CA, United States) at Biomarker Technologies (Beijing, China). Paired reads were filtered for quality (Q30) and joined by using FLASH version 1.2.11 (Magoc and Salzberg, 2011). Sequences that contained read lengths shorter than 400 bp were removed by using *PRINSEQ v0.20.4* (Schmieder and Edwards, 2011). The remaining sequences were then classified into operational taxonomic units (OTUs) by using QIIME 1.9.0 (Caporaso et al., 2010), at a 97% similarity threshold. OTUs, whose counts were more than 3 in at least one of the samples, were hierarchically summed at all taxonomic levels, and the counts were normalized to the relative abundance for each sample. The diversity of the microbial communities was estimated by using the R program phyloseq package (McMurdie and Holmes, 2013). The relationships between the abundance of each microbial genera and the concentrations of investigated SCFAs were explored by using constrained correspondence analysis (CCA) in the vegan package (Oksanen et al., 2016). The R program ggplot2 package (Wickham, 2009) was employed to generate the visual interpretation (biplot) of the SCFA-microbiota relationships. Finally, functional classifications were obtained by using PICRUSt 1.1.1, which predicts the Kyoto Encyclopedia of Genes and Genomes (KEGG) Ortholog groups for microbial 16S data.

### Database Submission

The sequencing data are available in the NCBI under BioProject PRJNA386408.

### Statistical Analyses

Data are expressed as means ± SE. Differences were considered significant when *P* < 0.05. The independent *t*-test (two-tailed test) was used in comparisons between two groups. Correlations between SCFA and UT-B mRNA abundance, between UT-B mRNA abundance and urinal urea-N excretion were calculated as Pearson correlation coefficients. All statistical analyses were performed by using SPSS (SPSS version 13.0.1 for Windows; SPSS Inc., Chicago, IL, United States) software packages.

## Results

### Effects of Dietary NFC on Rumen Characteristics and Serum Urea-N

Table 1 shows that, in the MNFC group, the SCFA concentration increased by 42% (106.50 ± 6.16 mM vs. 75.17 ± 4.49 mM, *p* < 0.01) compared with that in the LNFC group. The individual acids, such as acetic acid, propionic acid, and butyric acid increased by 36% (72.83 ± 6.17 mM vs. 53.43 ± 3.87 mM, *p* < 0.01), 62% (23.50 ± 4.69 mM vs. 14.50 ± 1.86 mM, *p* < 0.01), and 41% (10.17 ± 2.21 mM vs. 7.24 ± 1.35 mM, *p* < 0.05), respectively. The pH declined significantly (6.58 ± 0.07 vs. 6.82 ± 0.16, *p* < 0.05). The rumen NH_3_-N concentration was significantly higher by 60% (10.72 ± 0.71 mM vs. 6.68 ± 0.45 mM, *p* < 0.05) in the MNFC group than that in the LNFC group, and the ruminal liquid microbial protein concentration was enhanced by 31% (1.54 ± 0.12 g/L vs. 1.18 ± 0.05 g/L, *p* < 0.01) in the MNFC group. Nevertheless, the serum urea-N did not differ significantly between the groups (3.16 ± 0.11 mM vs. 3.39 ± 0.09 mM, *p* > 0.05).

**TABLE 1:** Effects of dietary NFC on rumen characteristics and serum urea-N concentration in experiment 1 (*n* = 10)^1^.

	**MNFC group**	**LNFC group**	***p-*value**
Rumen content			
SCFA concentrations (mM)			
Acetic acid	72.83 ± 6.17	53.43 ± 3.87	0.003
Propionic acid	23.50 ± 4.69	14.50 ± 1.86	0.002
Butyric acid	10.17 ± 2.21	7.24 ± 1.35	0.03
Total SCFA	106.5 ± 6.16	75.17 ± 4.49	0
pH	6.58 ± 0.07	6.82 ± 0.16	0.04
NH_3_-N (mM)	10.72 ± 0.71	6.68 ± 0.45	0
L-MCP (g/L)^2^	1.54 ± 0.12	1.18 ± 0.05	0.03
Serum urea-N (mM)	3.16 ± 0.11	3.39 ± 0.09	0.48

*^1^The intake of non-fiber carbohydrate was 28% in the MNFC group (*n* = 10) and 14% in the LNFC (*n* = 10) group; the intake of nitrogen was about equal in both groups. Analyses were performed in triplicate, except for the pH analysis, which was in duplicate. Values are means ± SE. *P* < 0.05 is considered significant. ^2^L-MCP: rumen liquid associated microbial protein.*

### Effects of Dietary NFC on Urea-N Excretion and Microbial Protein Synthesis

Urinal urea-N excretion was reduced by 37% (5.51 ± 0.37 mM/BW⋅kg/d vs. 8.71 ± 0.69 mM/BW⋅kg/d, *P* < 0.05, Table 2) in the MNFC group compared with that in the LNFC group. Microbial protein synthesis increased by 15% (3.71 ± 0.11 vs. 3.23 ± 0.05 g/d, *P* < 0.05, Table 2) in the MNFC group compared with that in the LNFC group.

**TABLE 2:** Effects of dietary NFC on urinal urea-N excretion and microbial protein synthesis in experiment 1 (*n* = 10)^1^.

	**MNFC group**	**LNFC group**	***p*-value**
Purine derivatives			
Allantoin (mM/d)	4.06 ± 0.12	3.53 ± 0.07	0.01
Uric acid (mM/d)	1.48 ± 0.05	1.28 ± 0.02	0.01
Microbial N (g/d)	3.71 ± 0.11	3.23 ± 0.05	0.004
UUE (mM/BW⋅kg/d)^2^	5.51 ± 0.37	8.71 ± 0.69	0.001

*^1^The intake of non-fiber carbohydrate was 28% in the MNFC group (*n* = 10) and 14% in the LNFC (*n* = 10) group; the intake of nitrogen was about equal in both groups. Analyses were performed in triplicate. Values are means ± SE. *P* < 0.05 is considered significant. ^2^UUE, urinal urea-N excretion.*

### Effects of Dietary NFC on Expression of UT-B and Receptors of SCFA in Rumen Epithelium

**FIGURE 1:**
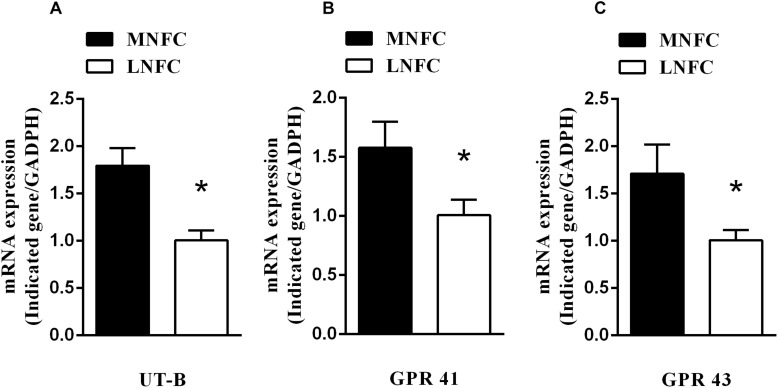
Effects of dietary NFC on **(A)** mRNA abundances of ruminal UT-B, **(B)** GPR 41, and **(C)** GPR 43 (*n* = 10). ^∗^Significant difference (*P* < 0.05) between treatments of MNFC and LNFC.

In the rumen epithelium, the mRNA abundance of UT-B increased by about 80% (*p* < 0.05, Figure 1A). This was accompanied by increases of GPR41 (about 60%, *p* < 0.05, Figure 1B) and GPR43 (about 70%, *p* < 0.05, Figure 1C).

The level of mRNA for UT-B was found to correlate positively with ruminal SCFA concentrations (*r* = 0.93, *P* < 0.05, Table 3). Moreover, the level of mRNA for UT-B was found to correlate negatively with urinal urea-N excretion (*r* = −0.76, *P* < 0.05, Table 3).

**TABLE 3:** Correlation between rumen epithelial UT-B mRNA and SCFA and between UT-B mRNA and urinal urea-N excretion (*n* = 20)^1^.

	**UT-B × SCFA**	**UT-B × UUE^2^**
Coefficient (*r*)	0.93	−0.72
*p*-value	0.00	0.01

*^1^Data were collected from Experiment 1 and calculated as Pearson’s correlation coefficients. ^2^Urinal urea-N excretion. *P* < 0.05 is considered significant.*

### Structure and Diversity of Microbial Communities

**FIGURE 2:**
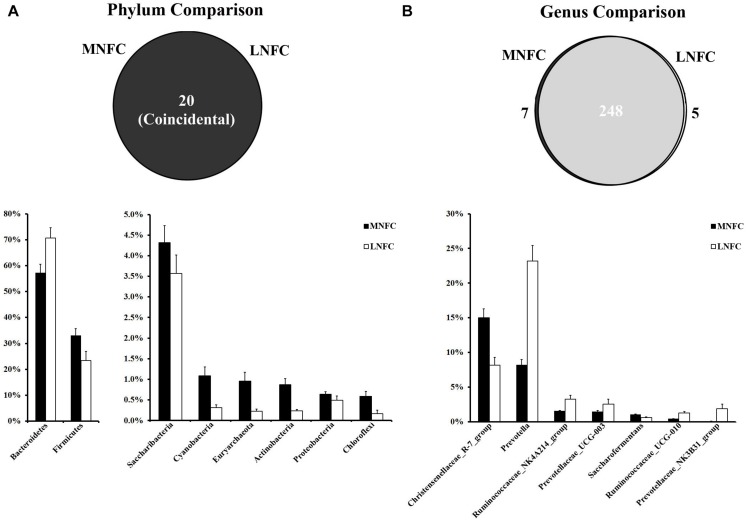
**(A)** Phylum-level comparison of bacterial OTUs between the MNFC group and the LNFC group. **(B)** Genus-level comparison of bacterial OTUs between the MNFC group and the LNFC group. Only the Phyla and Genera whose relative abundance was more than 1% in at least one of the samples are shown in the figure. MNFC (28% NFC):, LNFC (14% NFC).

At the phylum level, a total of 20 prokaryotic phyla were identified by a comparison with the GreenGene databases at a 97% similarity threshold. All of these phyla were common to both groups (Figure 2). Bacteroidetes (57–71%) and Firmicutes (33–23%) were most abundant in the two microbial communities. The relative abundances of all phyla in the two groups are shown in Supplementary Table S3. In the MNFC group, the relative abundances of Verrucomicrobia and Cyanobacteria (Figure 2) increased by 5.5 and 2.5 times more than that in the LNFC group, being the most expanded phyla. At the genus level, a total of 260 genera were detected in the sequences. Among them, 248 genera were common to both groups (Figure 2). The relative abundances of all genera in the two groups are shown in Supplementary Table S4. The Christensenellaceae R-7 group (15%) was the most abundant in the MNFC group, and *Prevotella* (8.25%) was the most abundant in the LNFC group. In the MNFC group, the relative abundance of *Lactobacillus* increased by 120% than that in the LNFC group, being the most expanded genus; contrarily, the relative abundances of *Carnobacterium* and *Psychrobacter* decreased by 99 and 98%, being the most shrunken genera. The relative abundances of all genera in the two groups are shown in Supplementary Table S3.

The Shannon and Simpson indices revealed that the diversity of microbial communities in the MNFC group was significantly higher than that in the LNFC group (*p* < 0.05) (Supplementary Figure S1).

### Correlation Between Abundance of Microbial Genera and SCFAs Concentrations

The effects of dietary NFC on rumen metabolites were consistent with those in Experiment 1 (Table 4). In the MNFC group, the SCFA concentrations increased by 43% (*p* < 0.01), and acetic acid, propionic acid, and butyric acid increased by 28% (*p* < 0.01), 57% (*p* < 0.01), and 94% (*p* < 0.01), respectively. However, pH was significantly lower (*p* < 0.05) in the MNFC group. Simultaneously, UT-B expression increased by 54% (*p* < 0.01).

**TABLE 4:** Effects of dietary NFC on ruminal SCFA, pH, and UT-B expression in experiment 2 (*n* = 4)^1,2^.

			**MNFC**	**LNFC**	***p*-value**
Rumen content	SCFA (mM)	Acetic acid	43.17 ± 2.1	33.79 ± 3.27	0.002
		Propionic acid	21.80 ± 1.40	13.86 ± 2.62	0.003
		Butyric acid	13.19 ± 2.44	6.80 ± 1.17	0.004
		Isobutyric acid	1.70 ± 0.10	1.13 ± 0.09	0.004
		Isopentanoic acid	1.71 ± 0.56	1.14 ± 0.16	0.057
		Pentanoic acid	1.53 ± 0.32	1.25 ± 0.19	0.128
		TSCA	83.10 ± 3.88	57.97 ± 3.27	0.001
	pH		6.53 ± 0.2	6.94 ± 0.08	0.02
Rumen epithelium	UT-B mRNA expression (Indicated gene/GADPH)		1.54 ± 0.08	1.01 ± 0.07	0.003

*^1^The intake of non-fiber carbohydrate was 28% in the MNFC group (*n* = 4) and 14% in the LNFC (*n* = 4) group; the intake of nitrogen was approximately equal in both groups. ^2^Analyses were performed in triplicate, except for pH analysis, which was in duplicate. Values are means ± SE. *P* < 0.05 is considered significant.*

**FIGURE 3:**
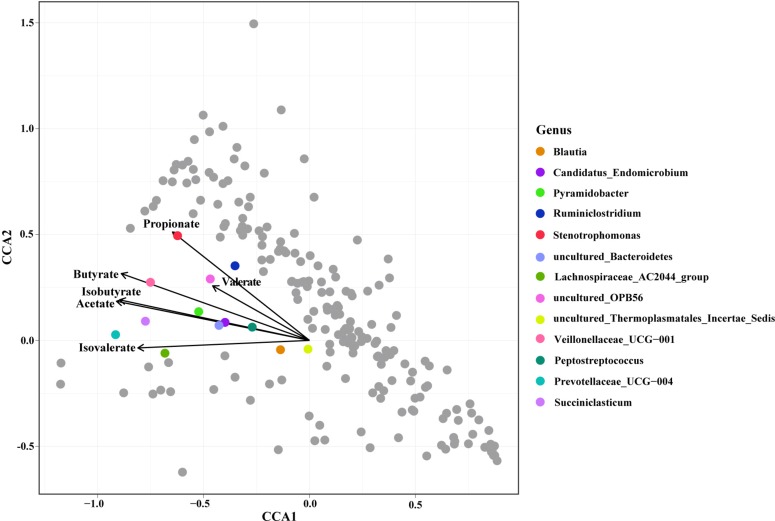
Constrained correspondence analysis revealing the correlations between the relative abundance of the microbial genus and the concentrations of ruminal Short Chain Fatty Acids (SCFAs).

Constrained correspondence analysis analysis revealed that the relative abundances of 13 genera were highly related to the SCFA concentrations (Figure 3). The concentration of Propionate was most related to the relative abundance of *Stenotrophomonas*. The concentration of Butyrate was most related to the relative abundance of *Veillonellaceae* UCG-001. The concentration of Acetate was highly related to the relative abundances of *Peptostreptococcus*, *Candidatus endomicrobium*, *Pyramidobacter*, and the uncultured species in Bacteroidetes. The SCFA concentration was highly related to the relative abundances of the *Blautia*, *Candidatus endomicrobium*, and the uncultured species in Bacteroidetes.

### Comparison of Microbial Function Between MNFC and LNFC Groups

**FIGURE 4:**
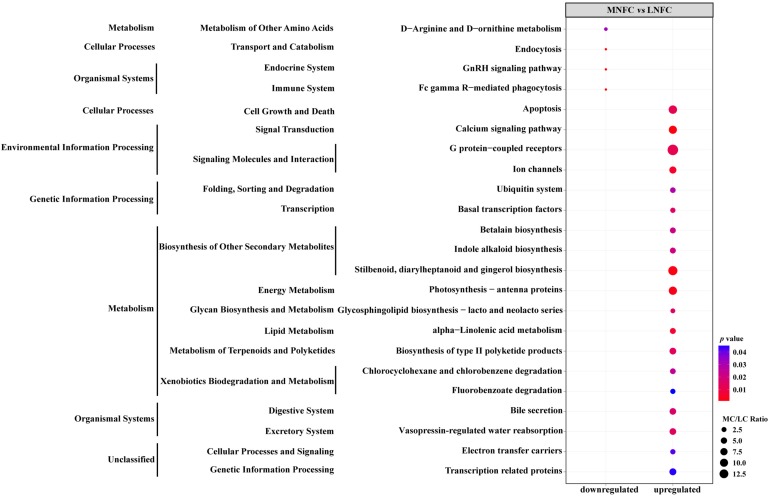
Variation trends of enriched Kyoto Encyclopedia of Genes and Genomes (KEGG) pathways in ruminal microbiota of the MNFC group compared with the LNFC group.

269 KEGG ortholog groups were detected in both of MNFC and LNFC groups (Supplementary Table S5). A comparison of the abundances of KEGG ortholog groups between the MNFC and LNFC groups showed that, in the MNFC group, 4 groups were significantly downregulated, whereas 19 groups were significantly upregulated (Figure 4).

## Discussion

### Ruminal SCFA and pH on Urinal Urea-N Excretion and Urea Transport Across Rumen Epithelium

In the present study, the goats in both groups received a diet of isonitrogenic, but differently proportioned NFC. The NFC proportion in the MNFC group was about double (28% vs. 14%) that in the LNFC group. The NFC-rich diet caused a reduction of urinal urea-N excretion by about 37%. This agrees with the report of Ma et al. who observed that, in lamb receiving a MNFC diet, the urinary N declined, but that fecal N did not change (Ma et al., 2015). Similarly, in growing steers, dietary carbohydrate facilitated urea being transferred from the blood directly to the rumen (Huntington, 1989; Younes et al., 2001).

In the present study, we have observed that, in goats consuming an NFC-rich diet, the concentration of ruminal NH_3_-N was enhanced significantly, together with a significantly reduced urinal urea-N excretion. These data indicate that more urea-N was transferred into the rumen under this feeding regime. In contrast, an infusion of urea into the rumen or a high level of dietary crude protein feeding caused the increase of the urinal urea-N excretion (Wickersham et al., 2008; Ahvenjarvi and Huhtanen, 2018). Taken together, the findings suggest that adequate dietary NFC feeding can improve nitrogen salvage in ruminant animals.

The effect of dietary NFC on serum urea-N is associated with rumen microbes, which use plant carbohydrate as a fermentation substrate to obtain energy for microbial growth and as an indirect supply to host animals. Among the composition of plant carbohydrate, lignin is known to be non-degradable in anaerobic environments (Triolo et al., 2012). Cellulose is more resistant to microbial fermentation, whereas soluble fiber (pectins and glucans) is less resistant, and grain, which is rich in starch, is easily fermentable. Dietary NFC, namely a fraction of easily-fermentable carbohydrate that includes starches, sugars, pectins, and glucans (Van Soest et al., 1991), leads to the rapid production of microbial metabolites, which are mainly SCFA (Hoover and Stokes, 1991). Previous studies have demonstrated that SCFA regulates blood urea entry into the rumen (Harmeyer and Martens, 1980; Remond et al., 2003). Remond et al. (2003) have reported that the highest rates of urea influx into the rumen and the highest NH_3_-N concentration in the rumen appears postprandially, when fermentation processes lead to a raise of SCFA in the rumen. Similarly, the present *in vivo* study has shown that, in the MNFC group, compared with the LNFC group, the serum urea-N concentration does not change, whereas the ruminal NH_3_-N concentration is enhanced, together with higher ruminal SCFA concentrations and a lower pH, indicating the altered ruminal SCFA and pH promoting liver-synthesized urea transferred from the blood to the rumen. The promoting effect of SCFA and pH on urea transport across the rumen epithelium has been observed in our previous *ex vivo* investigation (Lu et al., 2014) performed by means of the Ussing-chamber technique to measure urea flux in isolated rumen epithelium. Our results demonstrated that the application of SCFA in the buffer solution of the mucosal side (within 1 h) led to a dramatic increase of flux J_sm_^urea^, suggesting that ruminal SCFA rapidly promotes blood urea across the rumen epithelium. A decrease of pH from 7.4 to 6.6 in the mucosal buffer exerted the same effect on flux J_sm_^urea^ as SCFA did (Lu et al., 2014). These data are in good agreement with another previous *ex vivo* study (Abdoun et al., 2010; Lu et al., 2014) and are consistent with an earlier *in vivo* experiment (Harmeyer and Martens, 1980), providing supporting evidence that SCFA and pH stimulate blood urea entry into the rumen of goats.

To understand the mechanism whereby SCFA and pH promote blood urea entry into the rumen, we measured UT-B expression in the rumen epithelium in the feeding trial. In goats eating a NFC-rich diet, the UT-B expression increased significantly. Similar results have been reported in studies on Holstein heifers (Marini and Van Amburgh, 2003), goats (Lu et al., 2015) and cattle (Simmons et al., 2009) fed on an easily-fermentable diet. Our previously published work reported that the expression of UT-B mRNA and protein in rumen epithelial cells was promoted by increasing the SCFA concentration in the medium in primary cell culture. In the present study, the rumen SCFA concentration was higher in the MNFC group than that in the LNFC group. The statistical analysis showed a positive correlation between SCFA and UT-B, and a negative correlation between UT-B and urinal urea-N excretion.

In brief, the data from our previous and present studies suggest that ruminal SCFA stimulates the transport of blood urea into the rumen, thereby possibly leading to a decrease of urea from blood entering the kidney. This is, at least partly, related to the increase in UT-B expression. The promoting effects of SCFA on UT-B expression occurs via the binding of SCFA to their corresponding receptors in the target tissues (Shen et al., 2017). In the present study, the expression of SCFA receptors GPR43 and GPR41 increased in goats fed on a MNFC diet, indicating that the SCFA pathway is involved in the regulation of UT-B expression. It is probable that, a MNFC diet causes the enhancement of urea transport, and a reduction of urinal urea-N excretion is stimulated by SCFA and acidic pH.

### Effects of Dietary NFC on Ruminal Microbial Protein Synthesis

In ruminant animals, nitrogen salvage is related to urea recycling and ruminal NH_3_ utilization. In the rumen, NH_3__,_ the major end-product of protein degradation and urea hydrolysis, provides a source of N for MCP synthesis (Parker et al., 1995). In most species, the production of NH_3_ and its incorporation into microbial protein in the hindgut is considered of little nutritional benefit to the host because the microbial protein is involved in fecal formation. However, in ruminant animals, microbial protein supplies an average of about 60% (range of 34 to 89%) of the non-ammonia N, i.e., amino acid-N, protein-N, etc., flow to the duodenum (Clark et al., 1992).

Therefore, the pathway of N assimilation into microbial protein in the rumen is an essential component of protein flow to the small intestine and N retention in animals. We have studied the dietary effects on ruminal microbial protein synthesis by goats fed a diet containing 28 or 14% NFC. During the process of microbial protein synthesis, the NFC can rapidly provide energy to the rumen microflora for N capture (Rooke et al., 1987). In beef heifers fed on a low protein diet (10%), an increase in the ruminally easily-fermentable carbohydrate level can increase microbial N flow to the duodenum and improve the efficiency of the use of recycled urea-N for microbial protein synthesis (Davies et al., 2013). Hume (1970) has observed that SCFA increases the flow of total nitrogen from the rumen to the intestine. The present study has shown that microbial protein synthesis increases by 15% in goats consuming a diet containing of 28% NFC compared with 14% NFC. The increase of microbial protein synthesis in this study may be related to the synchronous release of NH_3_ and ruminal-avalaible energy via manipulation of urea transport and rumen fermentation, leading to an improvement of the efficiency of microbial N-transformation. This speculation is supported by a previous study (Sinclair et al., 1993). Our results indicate that NFC-intake-promoted microbial N-transformation is beneficial for nitrogen salvage and for decreasing urinal urea-N excretion. By contrast, an increase in dietary crud protein intake leads to a decrease in the fraction of recycled urea-N incorporated into microbial N (Batista et al., 2017).

In the present study in goats receiving a diet containing 28% of NFC, the ruminal NH3-N concentration was greater than that in goats receiving a diet containing 14% of NFC. In the rumen, the concentrations oscillations of NH_3_-N and microbial protein are not only affected by dietary protein and energy intake, but also by microbial metabolism. Previous studies have shown that increase of dietary protein and rumen available energy intake can induce synchronous increases of NH_3_-N concentration and microbial protein production in rumen of sheep (Ramos et al., 2009; Martínez et al., 2010). These authors speculated that the oscillation of NH_3_-N concentration may be attributable to different levels of N input in dietary composition change (Martínez et al., 2010). However, this was challenged by other researchers who observed an inconsistent response of NH_3_-N concentration to dietary intake. Granja-Salcedo et al. (2016) reported that cattle consumed an equal proportion of protein but different levels of NFC, the rumen microbial protein production increased with increasing NFC intake, but NH_3_-N concentration was decreased with this feeding (Granja-Salcedo et al., 2016). In another study, dietary starch provided to cattle receiving equal amount of protein resulted in greater microbial protein production, but no change in NH_3_-N concentration in the rumen (Owens et al., 2009). In the current study goats, received a diet containing the equal proportion of nitrogen but different levels of NFC their ruminal NH_3_-N concentration and ruminal microbial protein production increased synchronously in higher NFC intake goats.

The different responses to dietary treatments observed in the above studies can’t be explained by dietary N input. Based on their experiment and others’ earlier studies, Ahvenjarvi and Huhtanen’s (2018) suggested that rumen microbes can efficiently capture NH_3_-N from rumen fluid for their protein synthesis until sufficient intracellular NH_3_-N concentration is attained. After which the NH_3_-N concentrations started to increase in the extracellular rumen fluid. In the current study, the high-expression of UT-B may accelerate the entry of blood urea into the rumen, which probably facilitates the microbes to obtain sufficient intracellular NH_3_-N, leading to increase its concentration in rumen fluid. To this end, it can, at least partially, explain the increase in ruminal NH_3_-N concentration in the MNFC group.

### Effect of Dietary NFC on Rumen Microbiota Diversity and SCFA Producer

Our results show that all of the phyla and most of the genera of the microbiota are common to both groups. These data indicate rumen homeostasis and hence a healthy microbial community (Tan et al., 2014). This is also supported by observations in the present study. The increase of dietary NFC proportion from 14 to 28% provides chances for the colonization and expansion of microbes that ferment the easily-fermentable carbohydrate to SCFA in the rumen (Donaldson et al., 2016). In the MNFC group, the most expanded phyla are the Verrucomicrobia and Cyanobacteria (Supplementary Table S3). Bacteria of the Verrucomicrobia are known to contribute to the maintenance of the immune homeostasis in the rumen microbial community (Shen et al., 2016), and bacteria from the Cyanobacteria are associated with N fixation (Stewart, 1980). At the genus level, the *Christensenellaceae R-7* group was the most abundant in the MNFC group (Supplementary Table S4). Bacteria of the genus of *Christensenellaceae* exhibit a saccharolytic character. Their end products of glucose fermentation are acetic acid and a small amount of butyric acid (Morotomi et al., 2012). The increased abundance of *Christensenellaceae* positively correlates with body weight gain (Liu et al., 2015). In the MNFC group, the relative abundance of *Lactobacillus* increased and was the most expanded genus. *Lactobacillus*, acting as a probiotic, stimulates the immune system and promotes the growth and colonization of beneficial intestinal bacteria. Hence, it has many beneficial biological effects on human and calf health (Ren et al., 2014; Zhang et al., 2019) Contrarily, the relative abundances of Psychrobacter, with its enzymatic capacity of spoilage during the degradation of lipids, amino acids, and proteins (Broekaert et al., 2013), decreased by 98% in the MNFC group. These data show that a moderate increase of dietary NFC promotes beneficial ruminal bacteria, maintaining rumen homeostasis and fermenting the polysaccharides to generate SCFAs.

### Comparison of Microbial Function Between the MNFC and LNFC Groups

Function prediction of the microbial community showed that the types and abundance of metabolic genes, such as the genes that code for enzymes catalyzing amino acid and fatty acid metabolism, were upregulated by the MNFC diet, indicating a diversified and efficient metabolism occured in the microbiota in the MNFC group. The metabolism of the gut microbiota produces bioactive compounds, i.e., SCFA, trimethylamine, and endotoxin. These microbiota-derived metabolites signal to host organs and enable the gut bacteria to connect to the immune and hormone systems, to the host metabolism, and to other functions (Schroeder and Backhed, 2016). Dietary NFC changes the rumen microbiota composition and metabolism, making microbes a link between the diet and ruminal UT-B expression and microbial protein synthesis via their metabolite SCFA (Koh et al., 2016).

Taken together, our results have revealed that the mechanism underlying the easily-fermentable carbohydrate causes the reduction of urinal urea-N excretion in ruminant animals. Our data further indicate that an increase of NFC from 14 to 28% in the diet promotes the expansion and diversification of SCFA-producing microbes and the upregulation of genes encoding digestive enzymes in the microbial community. Such changes in the ruminal microbiota lead to an increase of the ruminal production of SCFA and, possibly, the expression of two SCFA receptors that might facilitate urea transport into the rumen, microbial protein synthesis, the reduction of urinal urea-N excretion. Interestingly, in lambs fed on a diet with higher levels of crude protein (15% vs. 10%) but with an equivalent carbohydrate composition, the concentrations of plasma urea-N and urinal urea-N excretion increased, but the blood urea-N entering the gut decreased. These results were associated with an unchanged SCFA concentration in the rumen and UT-B expression in the rumen epithelium (Kiran and Mutsvangwa, 2010).

As far as we know, this paper reports, for the first time, a comprehensive study of the interactions between dietary NFC and the rumen microbiota community, microbial fermentation, and rumen epithelial UT-B and GPRs and reveals the way that these interactions affect urinal urea-N excretion. We conclude that dietary-NFC-caused urea-N utilization is probably triggered by changes in microbiota diversity. The increase of SCFA producers and SCFA concentration in the rumen facilitates the transport of urea from the blood to the rumen and also increases microbial protein synthesis, leading to a reduction of urinal urea-N excretion. Our study thus provides new insights into the involvement of microbiota in dietary modulation; such insights should help to improve urea-N salvage in ruminant animals.

## Author Contributions

ZL and ZS designed the study. ZS wrote the manuscript. HS and ZL analyzed the data. ZL performed the experiments. All authors approved the final manuscript.

## Conflict of Interest Statement

The authors declare that the research was conducted in the absence of any commercial or financial relationships that could be construed as a potential conflict of interest.
