# Supplementary material for: Effects of Dietary-SCFA on Microbial Protein Synthesis and Urinal Urea-N Excretion Are Related to Microbiota Diversity in Rumen
Source: Front Physiol. 2019 Aug 22;10:1079. doi: 10.3389/fphys.2019.01079 (PMC6714491; doi:10.3389/fphys.2019.01079)
Supplement: Supplementary file 1 [file Data_Sheet_1.docx]

Supplementary Material

Effect of Dietary-SCFA on Microbial Protein Synthesis and Urinal Urea-N Excretion Is Related to Microbiota Diversity in Rumen

Zhongyan Lu^1#^, Hong Shen^2,3#^, Zanming Shen^1*^

**Correspondence:** Zanming Shen: [zmshen@njau.edu.cn](mailto:zmshen@njau.edu.cn)

# Supplementary Figures and Tables

## Supplementary Figure


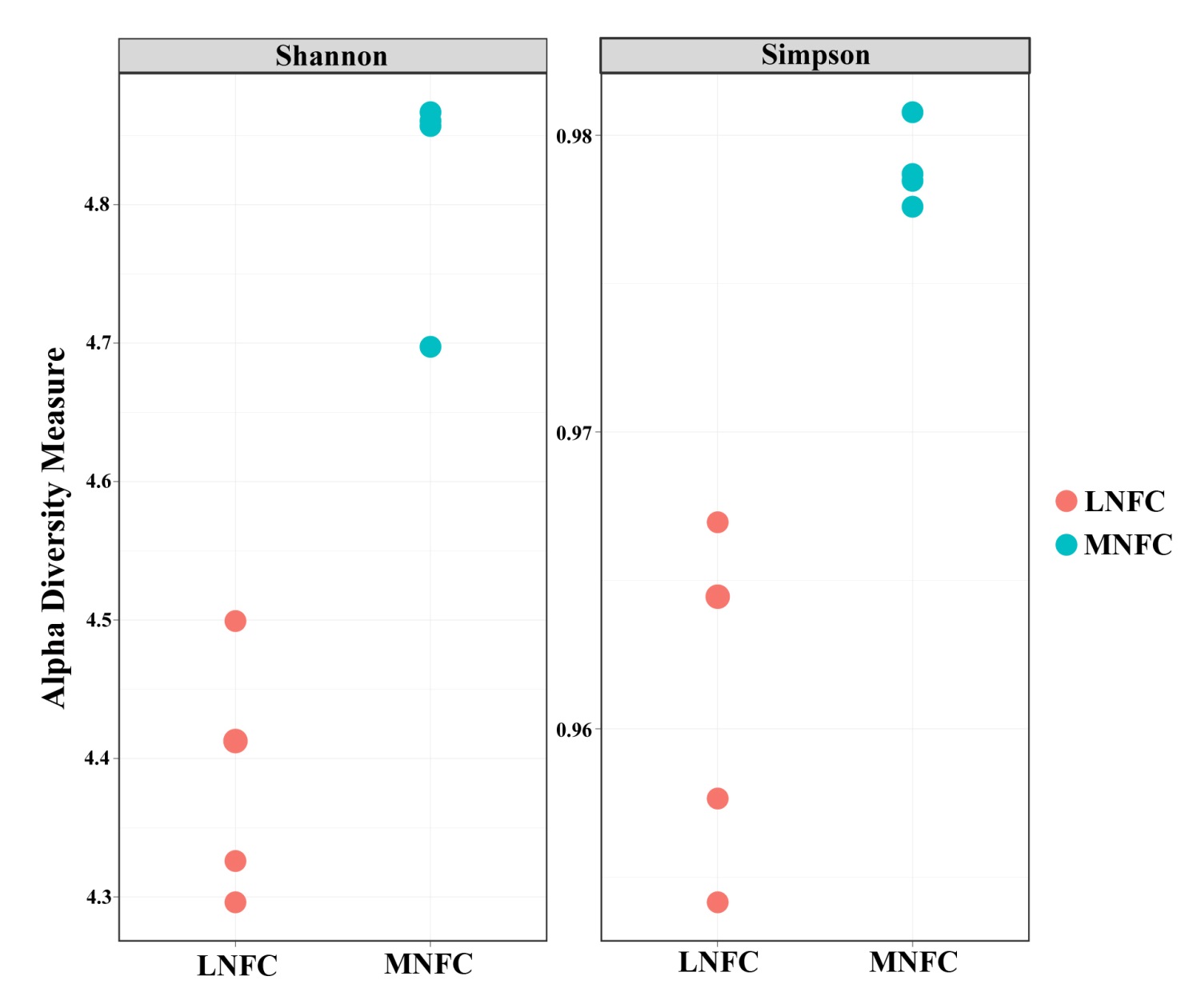
**Supplementary Figure 1**. Microbial diversity estimated by using Shannon and Simpson indices

## Supplementary Tables

**Supplementary Table 1**. Ingredient and chemical composition of diets in experiments

| Item | MNFC | LNFC |
| --- | --- | --- |
| **Ingredient, % of DM** |  |  |
| Guinea Grass | 70 | 92 |
| Ground Corn | 22 |  |
| Soya bean meal | 7 | 7 |
| Additive^2^ | 1 | 1 |
| **Chemical composition** |  |  |
| DM,% | 89.5 | 90.6 |
| Crude protein,%DM | 10.0 | 9.6 |
| Crude fat, %DM | 2.9 | 3.0 |
| Crude fibre, %DM | 22.8 | 29.5 |
| Crude ash, %DM | 5.2 | 6.0 |
| NDF, %DM | 53.6 | 67.3 |
| NFC^3^, %DM | 28.3 | 14.1 |
| ME,MJ/kg DM | 6.0 | 5.6 |

^1^ The values are means ± SE.

^2^ The additive was composed of calcium phosphate, limestone, trace mineral salt, and vitamin premix (vitamins A, D, and E)

^3^ NFC = 100 – (NDF + CP + crude fat + ash)

**Supplementary Table 2.** Oligonucleotide primers for target genes expressed in rumen epithelium

| Genes | Primmer sequence | Size  (bp) | Reference |
| --- | --- | --- | --- |
| GAPDH | Forward: 5- TTGTCTCCTGCGACTTCA -3  Reverse: 5- CCACCACCCTGTTACTGTT -3 | 167 | Genbank HM043737.1 |
| UT-B | Forward: 5-ggacctgcctgtcttcactc-3  Reverse: 5-GATCAAGGTGCTTGGGAAAA-3 | 97 | (Ludden et al., 2009)^1^ |
| GPR43 | Forward: 5-TTATCTTGCTCACCGGTCTC-3  Reverse: 5-TTCGATGATCTTGAAGGGCA-3 | 158 | Genbank NM_001163784.1 |
| GPR41 | Forward: 5- AAGTGGTCAAGGTGAGGAAG-3  Reverse: 5- CCAGCTTCGTCAGTAGTTCT-3 | 185 | Genbank NM_001285653 |

^1^Ludden PA, Stohrer RM, Austin KJ, Atkinson RL, Belden EL, Harlow HJ. Effect of protein supplementation on expression and distribution of urea transporter-B in lambs fed low-quality forage. J Anim Sci. 2009;87(4):1354-65.

**Supplementary Table 3.** Rumen microbial compositions of the MNFC and LNFC groups at the phylum level

| **Item** | **Phylum** | **LNFC** | **MNFC** |
| --- | --- | --- | --- |
| 1 | Bacteroidetes | 706795±40088 | 572547±32500 |
| 2 | Firmicutes | 234174±35078 | 330930±25798 |
| 3 | Saccharibacteria | 35668±4454 | 43231±4083 |
| 4 | Cyanobacteria | 3069±662 | 10895±2076 |
| 5 | Proteobacteria | 4940±1075 | 6412±587 |
| 6 | Tenericutes | 4282±828 | 1712±75 |
| 7 | Actinobacteria | 2434±333 | 8665±1548 |
| 8 | Euryarchaeota | 2285±502 | 9617±2135 |
| 9 | Chloroflexi | 1653±795 | 5935±1184 |
| 10 | Lentisphaerae | 1587±593 | 2544±634 |
| 11 | Spirochaetae | 1051±265 | 1016±88 |
| 12 | Verrucomicrobia | 755±189 | 4945±1639 |
| 13 | Elusimicrobia | 477±82 | 481±28 |
| 14 | Planctomycetes | 395±224 | 775±264 |
| 15 | Fibrobacteres | 155±45 | 211±46 |
| 16 | Fusobacteria | 133±20 | 13±5 |
| 17 | Chlorobi | 58±13 | 18±4 |
| 18 | SR1_(Absconditabacteria) | 57±20 | 27±10 |
| 19 | Microgenomates | 28±18 | 20±16 |
| 20 | Synergistetes | 18±15 | 5±5 |

Counts were normalized to the relative abundance of each sample and showed here in a scale of 1/1,000,000; genera were sorted based on their relative abundance in the rumen microbial community in a descending order; values are mean value ± SE (*n* = 4)

**Supplementary Table 4**. Rumen microbial compositions of MNFC and LNFC groups at the genus level

| **Item** | **Genus** | **LNFC** | **MNFC** |
| --- | --- | --- | --- |
| 1 | Rikenellaceae_RC9_gut_group | 280460±35064 | 374921±19236 |
| 2 | Prevotella | 231665±72482 | 82507±7384 |
| 3 | uncultured_Bacteroidales_BS11_gut_group | 114399±19593 | 86563±5656 |
| 4 | Christensenellaceae_R-7_group | 82034±10773 | 150033±13151 |
| 5 | Candidatus_Saccharimonas | 35660±4454 | 43212±4077 |
| 6 | Ruminococcaceae_NK4A214_group | 32844±5808 | 15736±597 |
| 7 | Prevotellaceae_UCG-003 | 25615±7223 | 15142±1357 |
| 8 | Prevotellaceae_NK3B31_group | 19198±6463 | 962±141 |
| 9 | uncultured_Bacteroidales_UCG-001 | 17542±5771 | 5903±575 |
| 10 | uncultured_Clostridiales_vadinBB60_group | 13061±2677 | 2980±329 |
| 11 | Ruminococcaceae_UCG-010 | 12813±2295 | 4563±274 |
| 12 | [Eubacterium]_coprostanoligenes_group | 10264±1762 | 10668±994 |
| 13 | Ruminococcus | 9598±1813 | 4945±274 |
| 14 | Ruminococcaceae_UCG-014 | 8546±1777 | 6147±502 |
| 15 | Saccharofermentans | 6235±1066 | 10852±403 |
| 16 | Phocaeicola | 6095±1572 | 1594±117 |
| 17 | Carnobacterium | 5658±887 | 72±28 |
| 18 | Lachnospiraceae_XPB1014_group | 3943±609 | 3996±176 |
| 19 | uncultured_Lachnospiraceae | 3418±547 | 5285±343 |
| 20 | Erysipelotrichaceae_UCG-009 | 3342±606 | 867±133 |
| 21 | Erysipelotrichaceae_UCG-004 | 3163±710 | 1870±177 |
| 22 | Prevotellaceae_UCG-001 | 3157±989 | 1254±84 |
| 23 | uncultured_Gastranaerophilales | 3029±645 | 10599±2086 |
| 24 | Butyrivibrio | 2780±413 | 2775±109 |
| 25 | [Eubacterium]_ventriosum_group | 2395±399 | 651±85 |
| 26 | Lachnospiraceae_UCG-002 | 2346±375 | 3286±215 |
| 27 | Ruminococcaceae_UCG-005 | 2303±343 | 680±41 |
| 28 | uncultured_NB1-n | 2211±364 | 161±26 |
| 29 | Methanobrevibacter | 2110±483 | 9312±2075 |
| 30 | uncultured_Bacteroidales_RF16_group | 1905±473 | 986±58 |
| 31 | Enterococcus | 1877±279 | 524±53 |
| 32 | Ruminiclostridium | 1750±147 | 582±47 |
| 33 | uncultured_Bacteroidales_S24-7_group | 1680±247 | 1162±67 |
| 34 | uncultured_Anaerolineaceae | 1632±805 | 5873±1175 |
| 35 | Lachnospiraceae_NK3A20_group | 1415±262 | 874±128 |
| 36 | uncultured_Porphyromonadaceae | 1393±238 | 558±60 |
| 37 | uncultured_Erysipelotrichaceae | 1353±292 | 3307±356 |
| 38 | uncultured_Mollicutes_RF9 | 1253±396 | 549±108 |
| 39 | uncultured_Ruminococcaceae | 1201±167 | 1463±145 |
| 40 | Roseburia | 1193±180 | 3553±240 |
| 41 | Lachnospiraceae_UCG-006 | 1162±215 | 558±42 |
| 42 | Oribacterium | 1149±163 | 134±16 |
| 43 | Howardella | 1143±154 | 1710±137 |
| 44 | uncultured_Rhodospirillaceae | 1142±553 | 428±11 |
| 45 | uncultured_vadinBE97 | 1127±401 | 2067±498 |
| 46 | Alloprevotella | 1121±378 | 263±28 |
| 47 | Succiniclasticum | 974±317 | 383±17 |
| 48 | Treponema | 941±280 | 799±88 |
| 49 | Lachnospiraceae_UCG-008 | 936±152 | 906±77 |
| 50 | Acetitomaculum | 920±178 | 1185±201 |
| 51 | Family_XIII_AD3011_group | 892±108 | 1239±174 |
| 52 | uncultured_ODP1230B8.23 | 815±122 | 44±8 |
| 53 | Anaerovorax | 754±153 | 471±62 |
| 54 | uncultured_Coriobacteriaceae | 730±158 | 1680±242 |
| 55 | Anaeroplasma | 723±133 | 792±70 |
| 56 | Psychrobacter | 708±76 | 8±5 |
| 57 | Ruminococcaceae_UCG-002 | 706±129 | 262±22 |
| 58 | uncultured_WCHB1-41 | 702±188 | 4930±1637 |
| 59 | uncultured_Christensenellaceae | 663±89 | 1313±113 |
| 60 | Syntrophococcus | 651±116 | 687±29 |
| 61 | uncultured_Clostridiales | 648±124 | 13±5 |
| 62 | Lactobacillus | 617±89 | 74800±7955 |
| 63 | Pseudobutyrivibrio | 603±100 | 675±70 |
| 64 | Ruminococcaceae_V9D2013_group | 594±94 | 127±19 |
| 65 | Prevotellaceae_UCG-004 | 573±123 | 61±11 |
| 66 | Bacteroides | 512±81 | 72±20 |
| 67 | Papillibacter | 478±38 | 633±20 |
| 68 | Ruminococcaceae_UCG-001 | 476±84 | 417±45 |
| 69 | uncultured_Victivallaceae | 460±196 | 477±139 |
| 70 | Anaerosporobacter | 452±88 | 7±7 |
| 71 | Pelagibacterium | 444±63 | 210±23 |
| 72 | Senegalimassilia | 436±56 | 3836±924 |
| 73 | Ruminococcaceae_UCG-013 | 410±82 | 21±10 |
| 74 | Lachnospiraceae_AC2044_group | 359±38 | 87±21 |
| 75 | [Ruminococcus]_gauvreauii_group | 354±44 | 476±44 |
| 76 | Atopobium | 352±35 | 1145±232 |
| 77 | Mogibacterium | 336±41 | 1896±283 |
| 78 | Elusimicrobium | 335±40 | 423±27 |
| 79 | Desulfovibrio | 330±49 | 246±13 |
| 80 | uncultured_Phyllobacteriaceae | 328±37 | 288±43 |
| 81 | uncultured_Peptococcaceae | 286±40 | 363±32 |
| 82 | Porphyromonas | 285±63 | 44±11 |
| 83 | Veillonellaceae_UCG-001 | 284±90 | 26±6 |
| 84 | uncultured_Ruminococcaceae | 267±30 | 244±29 |
| 85 | Petrimonas | 253±50 | 25±12 |
| 86 | Olsenella | 246±79 | 201±43 |
| 87 | Clostridium_sensu_stricto3 | 229±30 | 0±0 |
| 88 | Ruminococcaceae_UCG-004 | 226±35 | 868±31 |
| 89 | Lachnospiraceae_FCS020_group | 224±38 | 99±27 |
| 90 | uncultured_Family_XIII | 221±51 | 3±3 |
| 91 | uncultured_Coriobacteriaceae | 218±43 | 446±44 |
| 92 | Anaerofustis | 217±48 | 578±92 |
| 93 | Devosia | 215±48 | 151±13 |
| 94 | Pseudochrobactrum | 201±38 | 295±40 |
| 95 | Prevotellaceae_YAB2003_group | 196±47 | 65±16 |
| 96 | Clostridium_sensu_stricto | 186±74 | 330±84 |
| 97 | [Eubacterium]_nodatum_group | 179±45 | 113±21 |
| 98 | uncultured_Rhodobacteraceae | 173±24 | 73±32 |
| 99 | Pirellula | 165±120 | 236±77 |
| 100 | Anaerotruncus | 161±33 | 29±13 |
| 101 | Methylobacterium | 158±39 | 242±22 |
| 102 | Solobacterium | 157±64 | 497±58 |
| 103 | Fibrobacter | 155±45 | 211±46 |
| 104 | Stenotrophomonas | 154±27 | 19±10 |
| 105 | Tyzzerella | 153±26 | 3±3 |
| 106 | Citrobacter | 147±21 | 19±7 |
| 107 | Lachnospiraceae_NK4A136_group | 143±21 | 53±9 |
| 108 | uncultured_Erysipelotrichaceae | 143±30 | 39±9 |
| 109 | Candidatus_Endomicrobium | 142±44 | 58±7 |
| 110 | uncultured_Marinilabiaceae | 135±37 | 20±5 |
| 111 | p-1088-a5_gut_group | 128±66 | 384±170 |
| 112 | Lachnoclostridium_5 | 122±33 | 6±6 |
| 113 | U29-B03 | 116±51 | 191±24 |
| 114 | Defluviitaleaceae_UCG-011 | 115±15 | 79±5 |
| 115 | Corynebacterium | 112±16 | 125±31 |
| 116 | Spirochaeta | 111±23 | 202±21 |
| 117 | uncultured_Bacteroidetes_BD2-2 | 104±23 | 17±5 |
| 118 | Fusobacterium | 103±27 | 13±5 |
| 119 | Moheibacter | 98±15 | 28±4 |
| 120 | Myroides | 94±15 | 0±0 |
| 121 | Erysipelotrichaceae_UCG-006 | 90±20 | 110±28 |
| 122 | Methanosphaera | 89±33 | 211±52 |
| 123 | Aminobacter | 89±14 | 99±6 |
| 124 | Sphingomonas | 88±19 | 33±7 |
| 125 | uncultured_Thermoplasmatales_IS | 86±19 | 94±15 |
| 126 | Sphingobacterium | 86±35 | 16±6 |
| 127 | Vagococcus | 86±17 | 3±3 |
| 128 | Halomonas | 84±32 | 2876±556 |
| 129 | Marvinbryantia | 77±14 | 49±17 |
| 130 | Lysinibacillus | 72±9 | 0±0 |
| 131 | Camelimonas | 71±25 | 63±7 |
| 132 | Comamonas | 69±10 | 45±7 |
| 133 | Alcaligenes | 69±25 | 35±12 |
| 134 | uncultured_Family_XI | 69±21 | 3±3 |
| 135 | [Eubacterium]_brachy_group | 66±17 | 56±2 |
| 136 | CPla-4_termite_group | 64±35 | 124±41 |
| 137 | Paracoccus | 64±19 | 6±4 |
| 138 | Shinella | 61±10 | 125±10 |
| 139 | Lysinimicrobium | 61±22 | 49±8 |
| 140 | Prevotellaceae_Ga6A1_group | 60±14 | 66±22 |
| 141 | Guggenheimella | 60±17 | 0±0 |
| 142 | uncultured_Mycoplasmataceae | 58±24 | 76±16 |
| 143 | uncultured_OPB56 | 58±13 | 18±4 |
| 144 | Erysipelotrichaceae_UCG-008 | 57±9 | 129±5 |
| 145 | uncultured_SR1_(Absconditabacteria) | 57±20 | 27±10 |
| 146 | Blautia | 56±7 | 40±15 |
| 147 | [Eubacterium]_ruminantium_group | 55±17 | 14±3 |
| 148 | Pediococcus | 54±10 | 60±16 |
| 149 | uncultured_OPB35_soil_group | 53±12 | 15±6 |
| 150 | Coriobacteriaceae_UCG-002 | 49±10 | 114±17 |
| 151 | Brevundimonas | 49±10 | 85±24 |
| 152 | Oscillospira | 48±14 | 414±20 |
| 153 | Cryobacterium | 48±8 | 57±17 |
| 154 | Leucobacter | 48±7 | 55±19 |
| 155 | uncultured_SAR324_clade(Marine_group_B) | 44±14 | 110±28 |
| 156 | uncultured_Oligoflexaceae | 42±12 | 460±23 |
| 157 | uncultured_Chloroplast | 41±19 | 297±34 |
| 158 | Family_XIII_UCG-002 | 40±11 | 5±3 |
| 159 | Enterorhabdus | 38±13 | 536±126 |
| 160 | Peptoniphilus | 38±9 | 0±0 |
| 161 | uncultured_NED5E9 | 37±8 | 135±11 |
| 162 | Candidatus_Soleaferrea | 37±4 | 45±21 |
| 163 | [Eubacterium]_hallii_group | 36±12 | 32±13 |
| 164 | Adlercreutzia | 35±12 | 41±12 |
| 165 | Lachnospiraceae_ND3007_group | 35±13 | 34±10 |
| 166 | Lachnospiraceae_FE2018_group | 35±15 | 22±10 |
| 167 | Erysipelothrix | 35±18 | 0±0 |
| 168 | [Eubacterium]_saphenum_group | 34±5 | 179±39 |
| 169 | uncultured_PeH15 | 34±16 | 53±24 |
| 170 | Streptococcus | 30±8 | 514±57 |
| 171 | Succinivibrio | 30±13 | 61±14 |
| 172 | Caviibacter | 30±13 | 0±0 |
| 173 | uncultured_Candidatus_Pacebacteria | 28±18 | 20±16 |
| 174 | Ruminiclostridium_5 | 28±13 | 17±8 |
| 175 | Azospirillum | 28±8 | 14±3 |
| 176 | Peptostreptococcus | 27±8 | 14±9 |
| 177 | Slackia | 26±13 | 35±9 |
| 178 | Lachnoclostridium0 | 26±16 | 24±11 |
| 179 | uncultured_vadinHA49 | 26±10 | 6±3 |
| 180 | Bosea | 23±12 | 20±5 |
| 181 | Bordetella | 22±9 | 7±7 |
| 182 | Luteimonas | 22±16 | 5±5 |
| 183 | uncultured_vadinBA26 | 21±15 | 62±10 |
| 184 | uncultured_Family_XIII | 21±3 | 50±20 |
| 185 | Trueperella | 21±3 | 32±8 |
| 186 | Tabrizicola | 21±12 | 27±10 |
| 187 | Paenochrobactrum | 20±10 | 24±8 |
| 188 | Desulfobulbus | 18±4 | 59±15 |
| 189 | Proteiniphilum | 18±2 | 30±8 |
| 190 | Pyramidobacter | 18±15 | 5±5 |
| 191 | uncultured_Lachnospiraceae | 14±5 | 28±7 |
| 192 | Pseudomonas | 13±8 | 19±11 |
| 193 | Termite_planctomycete_cluster | 12±9 | 25±9 |
| 194 | Intestinibacter | 11±4 | 771±84 |
| 195 | Denitrobacterium | 10±6 | 152±43 |
| 196 | Acinetobacter | 8±5 | 52±15 |
| 197 | Bacillus | 8±5 | 51±14 |
| 198 | uncultured_Saccharibacteria | 7±2 | 20±10 |
| 199 | Succinivibrionaceae_UCG-002 | 6±6 | 94±14 |
| 200 | Nesterenkonia | 5±5 | 164±59 |
| 201 | Cloacibacterium | 5±5 | 45±11 |
| 202 | Turicibacter | 3±3 | 572±113 |
| 203 | uncultured_Peptostreptococcaceae | 3±3 | 475±56 |
| 204 | uncultured_Mitochondria | 3±3 | 42±5 |
| 205 | Ruminobacter | 0±0 | 76±9 |
| 206 | Quinella | 0±0 | 62±7 |
| 207 | Ruminiclostridium_9 | 0±0 | 46±5 |
| 208 | Lachnospiraceae_UCG-001 | 0±0 | 44±9 |
| 209 | Sphaerochaeta | 0±0 | 15±11 |

Counts were normalized to the relative abundance of each sample and showed here in a scale of 1/1,000,000; genera were sorted based on their relative abundance in the rumen microbial community in a descending order; values are mean value ± SE (*n* = 4)

**Supplementary Table 5**. Comparison of KEGG ortholog abundances between MNFC group and LNFC group

|  | KEGG_level_1 | KEGG_level_2 | LNFC | MNFC | MSE |
| --- | --- | --- | --- | --- | --- |
| Cellular Processes | Cell Growth and Death | Apoptosis | 283 | 1978 | 156 |
|  | Cell Growth and Death | Cell cycle | 103 | 411 | 53 |
|  | Cell Growth and Death | Cell cycle - Caulobacter | 234232 | 243672 | 15421 |
|  | Cell Growth and Death | Meiosis - yeast | 2531 | 2890 | 237 |
|  | Cell Growth and Death | p53 signaling pathway | 256 | 129 | 40 |
|  | Cell Motility | Bacterial chemotaxis | 143273 | 166504 | 15705 |
|  | Cell Motility | Bacterial motility proteins | 279255 | 325151 | 31293 |
|  | Cell Motility | Cytoskeleton proteins | 136606 | 150930 | 10134 |
|  | Cell Motility | Flagellar assembly | 118820 | 137434 | 12547 |
|  | Transport and Catabolism | Endocytosis | 13 | 1 | 0 |
|  | Transport and Catabolism | Lysosome | 75984 | 90474 | 7447 |
|  | Transport and Catabolism | Peroxisome | 101025 | 108154 | 6953 |
|  | Transport and Catabolism | Phagosome | 103 | 411 | 53 |
| Environmental Information Processing | Membrane Transport | ABC transporters | 844028 | 997580 | 76785 |
|  | Membrane Transport | Bacterial secretion system | 257838 | 270207 | 17132 |
|  | Membrane Transport | Phosphotransferase system (PTS) | 63881 | 85323 | 9266 |
|  | Membrane Transport | Secretion system | 423025 | 464111 | 30189 |
|  | Membrane Transport | Transporters | 1724880 | 1997727 | 151782 |
|  | Signal Transduction | Calcium signaling pathway | 2 | 13 | 1 |
|  | Signal Transduction | MAPK signaling pathway - yeast | 27945 | 32837 | 2229 |
|  | Signal Transduction | mTOR signaling pathway | 103 | 411 | 53 |
|  | Signal Transduction | Phosphatidylinositol signaling system | 35900 | 41371 | 2773 |
|  | Signal Transduction | Two-component system | 450378 | 521900 | 37876 |
|  | Signaling Molecules and Interaction | Bacterial toxins | 37200 | 45717 | 3056 |
|  | Signaling Molecules and Interaction | Cellular antigens | 34359 | 30060 | 3056 |
|  | Signaling Molecules and Interaction | G protein-coupled receptors | 1 | 13 | 1 |
|  | Signaling Molecules and Interaction | Ion channels | 1276 | 5485 | 301 |
| Genetic Information Processing | Folding, Sorting and Degradation | Chaperones and folding catalysts | 464386 | 489186 | 31269 |
|  | Folding, Sorting and Degradation | Proteasome | 21011 | 22574 | 1317 |
|  | Folding, Sorting and Degradation | Protein export | 285536 | 301258 | 19050 |
|  | Folding, Sorting and Degradation | Protein processing in endoplasmic reticulum | 42269 | 44785 | 2525 |
|  | Folding, Sorting and Degradation | RNA degradation | 217805 | 235152 | 14766 |
|  | Folding, Sorting and Degradation | Sulfur relay system | 77670 | 94855 | 7062 |
|  | Folding, Sorting and Degradation | Ubiquitin system | 239 | 488 | 45 |
|  | Replication and Repair | Base excision repair | 181134 | 201179 | 12308 |
|  | Replication and Repair | Chromosome | 662945 | 704070 | 45081 |
|  | Replication and Repair | DNA repair and recombination proteins | 1254867 | 1335126 | 84198 |
|  | Replication and Repair | DNA replication | 309184 | 330798 | 21213 |
|  | Replication and Repair | DNA replication proteins | 571913 | 613878 | 38844 |
|  | Replication and Repair | Homologous recombination | 435831 | 461160 | 29858 |
|  | Replication and Repair | Mismatch repair | 369165 | 393682 | 25232 |
|  | Replication and Repair | Non-homologous end-joining | 2011 | 1807 | 200 |
|  | Replication and Repair | Nucleotide excision repair | 176987 | 191233 | 11758 |
|  | Transcription | Basal transcription factors | 2186 | 3606 | 198 |
|  | Transcription | RNA polymerase | 71636 | 80503 | 4613 |
|  | Transcription | Transcription factors | 457099 | 514815 | 39230 |
|  | Transcription | Transcription machinery | 427731 | 449322 | 28444 |
|  | Translation | Aminoacyl-tRNA biosynthesis | 518666 | 562930 | 34761 |
|  | Translation | mRNA surveillance pathway | 207 | 822 | 105 |
|  | Translation | Ribosome | 1149394 | 1230276 | 75988 |
|  | Translation | Ribosome Biogenesis | 602785 | 650619 | 41627 |
|  | Translation | Ribosome biogenesis in eukaryotes | 22475 | 26365 | 1381 |
|  | Translation | RNA transport | 52550 | 62298 | 4152 |
|  | Translation | Translation factors | 264224 | 285358 | 17653 |
| Human Diseases | Cancers | Bladder cancer | 88 | 119 | 15 |
|  | Cancers | Colorectal cancer | 255 | 129 | 40 |
|  | Cancers | Pathways in cancer | 20465 | 20726 | 1345 |
|  | Cancers | Prostate cancer | 18993 | 19527 | 1297 |
|  | Cancers | Renal cell carcinoma | 1226 | 1071 | 95 |
|  | Cancers | Small cell lung cancer | 255 | 129 | 40 |
|  | Cardiovascular Diseases | Hypertrophic cardiomyopathy (HCM) | 6 | 3 | 1 |
|  | Cardiovascular Diseases | Viral myocarditis | 255 | 129 | 40 |
|  | Immune System Diseases | Primary immunodeficiency | 16162 | 17745 | 1235 |
|  | Immune System Diseases | Systemic lupus erythematosus | 9 | 16 | 4 |
|  | Infectious Diseases | African trypanosomiasis | 990 | 841 | 88 |
|  | Infectious Diseases | Amoebiasis | 1541 | 1367 | 141 |
|  | Infectious Diseases | Bacterial invasion of epithelial cells | 21 | 17 | 2 |
|  | Infectious Diseases | Chagas disease (American trypanosomiasis) | 816 | 530 | 92 |
|  | Infectious Diseases | Epithelial cell signaling in Helicobacter pylori infection | 40081 | 41841 | 2662 |
|  | Infectious Diseases | Hepatitis C | 103 | 411 | 53 |
|  | Infectious Diseases | Influenza A | 358 | 540 | 73 |
|  | Infectious Diseases | Measles | 103 | 411 | 53 |
|  | Infectious Diseases | Pertussis | 13271 | 12591 | 1177 |
|  | Infectious Diseases | Toxoplasmosis | 255 | 129 | 40 |
|  | Infectious Diseases | Tuberculosis | 67473 | 74364 | 4592 |
|  | Infectious Diseases | Vibrio cholerae infection | 104 | 411 | 53 |
|  | Infectious Diseases | Vibrio cholerae pathogenic cycle | 29664 | 29666 | 2037 |
|  | Metabolic Diseases | Type I diabetes mellitus | 29155 | 33415 | 2239 |
|  | Metabolic Diseases | Type II diabetes mellitus | 22231 | 23427 | 1500 |
|  | Neurodegenerative Diseases | Alzheimer's disease | 23177 | 23696 | 1607 |
|  | Neurodegenerative Diseases | Amyotrophic lateral sclerosis (ALS) | 8685 | 11995 | 1075 |
|  | Neurodegenerative Diseases | Huntington's disease | 18240 | 20197 | 1238 |
|  | Neurodegenerative Diseases | Parkinson's disease | 804 | 507 | 146 |
|  | Neurodegenerative Diseases | Prion diseases | 208 | 252 | 28 |
| Metabolism | Amino Acid Metabolism | Alanine, aspartate and glutamate metabolism | 497870 | 518673 | 32787 |
|  | Amino Acid Metabolism | Amino acid related enzymes | 658240 | 700910 | 43680 |
|  | Amino Acid Metabolism | Arginine and proline metabolism | 543943 | 584010 | 36650 |
|  | Amino Acid Metabolism | Cysteine and methionine metabolism | 405489 | 427898 | 26583 |
|  | Amino Acid Metabolism | Glycine, serine and threonine metabolism | 395736 | 410760 | 26118 |
|  | Amino Acid Metabolism | Histidine metabolism | 286577 | 309559 | 19860 |
|  | Amino Acid Metabolism | Lysine biosynthesis | 356615 | 380363 | 24310 |
|  | Amino Acid Metabolism | Lysine degradation | 52551 | 61301 | 4307 |
|  | Amino Acid Metabolism | Phenylalanine metabolism | 84459 | 83863 | 5120 |
|  | Amino Acid Metabolism | Phenylalanine, tyrosine and tryptophan biosynthesis | 401525 | 411640 | 25793 |
|  | Amino Acid Metabolism | Tryptophan metabolism | 72514 | 86936 | 6248 |
|  | Amino Acid Metabolism | Tyrosine metabolism | 132190 | 145890 | 9057 |
|  | Amino Acid Metabolism | Valine, leucine and isoleucine biosynthesis | 331617 | 356555 | 22641 |
|  | Amino Acid Metabolism | Valine, leucine and isoleucine degradation | 114131 | 129448 | 7771 |
|  | Biosynthesis of Other Secondary Metabolites | beta-Lactam resistance | 8536 | 11046 | 1015 |
|  | Biosynthesis of Other Secondary Metabolites | Betalain biosynthesis | 79 | 194 | 26 |
|  | Biosynthesis of Other Secondary Metabolites | Butirosin and neomycin biosynthesis | 38969 | 41271 | 2738 |
|  | Biosynthesis of Other Secondary Metabolites | Caffeine metabolism | 19 | 16 | 2 |
|  | Biosynthesis of Other Secondary Metabolites | Flavone and flavonol biosynthesis | 457 | 416 | 82 |
|  | Biosynthesis of Other Secondary Metabolites | Flavonoid biosynthesis | 1104 | 1775 | 170 |
|  | Biosynthesis of Other Secondary Metabolites | Indole alkaloid biosynthesis | 78 | 193 | 26 |
|  | Biosynthesis of Other Secondary Metabolites | Isoflavonoid biosynthesis | 24 | 112 | 20 |
|  | Biosynthesis of Other Secondary Metabolites | Isoquinoline alkaloid biosynthesis | 28327 | 27653 | 1899 |
|  | Biosynthesis of Other Secondary Metabolites | Novobiocin biosynthesis | 65563 | 69969 | 4218 |
|  | Biosynthesis of Other Secondary Metabolites | Penicillin and cephalosporin biosynthesis | 9957 | 12354 | 1069 |
|  | Biosynthesis of Other Secondary Metabolites | Phenylpropanoid biosynthesis | 85296 | 76921 | 6681 |
|  | Biosynthesis of Other Secondary Metabolites | Stilbenoid, diarylheptanoid and gingerol biosynthesis | 274 | 2404 | 141 |
|  | Biosynthesis of Other Secondary Metabolites | Streptomycin biosynthesis | 160130 | 172185 | 10996 |
|  | Biosynthesis of Other Secondary Metabolites | Tropane, piperidine and pyridine alkaloid biosynthesis | 54012 | 55394 | 3459 |
|  | Carbohydrate Metabolism | Amino sugar and nucleotide sugar metabolism | 571773 | 613399 | 39132 |
|  | Carbohydrate Metabolism | Ascorbate and aldarate metabolism | 37948 | 38146 | 2729 |
|  | Carbohydrate Metabolism | Butanoate metabolism | 264505 | 296617 | 19171 |
|  | Carbohydrate Metabolism | C5-Branched dibasic acid metabolism | 131411 | 144762 | 9543 |
|  | Carbohydrate Metabolism | Citrate cycle (TCA cycle) | 335762 | 356017 | 21751 |
|  | Carbohydrate Metabolism | Fructose and mannose metabolism | 343920 | 349132 | 22937 |
|  | Carbohydrate Metabolism | Galactose metabolism | 303481 | 329785 | 22333 |
|  | Carbohydrate Metabolism | Glycolysis / Gluconeogenesis | 447830 | 480942 | 30864 |
|  | Carbohydrate Metabolism | Glyoxylate and dicarboxylate metabolism | 205744 | 216524 | 13280 |
|  | Carbohydrate Metabolism | Inositol phosphate metabolism | 39374 | 44909 | 3039 |
|  | Carbohydrate Metabolism | Pentose and glucuronate interconversions | 215391 | 209280 | 14499 |
|  | Carbohydrate Metabolism | Pentose phosphate pathway | 299766 | 320605 | 21599 |
|  | Carbohydrate Metabolism | Propanoate metabolism | 202004 | 227648 | 15201 |
|  | Carbohydrate Metabolism | Pyruvate metabolism | 421972 | 472263 | 31027 |
|  | Carbohydrate Metabolism | Starch and sucrose metabolism | 402709 | 401940 | 26831 |
|  | Energy Metabolism | Carbon fixation in photosynthetic organisms | 272715 | 279467 | 17745 |
|  | Energy Metabolism | Carbon fixation pathways in prokaryotes | 497056 | 533699 | 32862 |
|  | Energy Metabolism | Methane metabolism | 536485 | 623385 | 37309 |
|  | Energy Metabolism | Nitrogen metabolism | 289029 | 297708 | 18759 |
|  | Energy Metabolism | Oxidative phosphorylation | 580117 | 633166 | 38981 |
|  | Energy Metabolism | Photosynthesis | 165169 | 178910 | 11193 |
|  | Energy Metabolism | Photosynthesis - antenna proteins | 33 | 218 | 24 |
|  | Energy Metabolism | Photosynthesis proteins | 165458 | 179558 | 11193 |
|  | Energy Metabolism | Sulfur metabolism | 99612 | 100134 | 6201 |
|  | Enzyme Families | Peptidases | 809238 | 864500 | 54544 |
|  | Enzyme Families | Protein kinases | 84694 | 95769 | 7137 |
|  | Glycan Biosynthesis and Metabolism | Glycosaminoglycan degradation | 56633 | 66383 | 5254 |
|  | Glycan Biosynthesis and Metabolism | Glycosphingolipid biosynthesis - ganglio series | 48001 | 54680 | 4323 |
|  | Glycan Biosynthesis and Metabolism | Glycosphingolipid biosynthesis - globo series | 77306 | 88366 | 6625 |
|  | Glycan Biosynthesis and Metabolism | Glycosphingolipid biosynthesis - lacto and neolacto series | 1 | 1 | 0 |
|  | Glycan Biosynthesis and Metabolism | Glycosyltransferases | 144422 | 141858 | 9532 |
|  | Glycan Biosynthesis and Metabolism | Lipopolysaccharide biosynthesis | 164443 | 157436 | 13144 |
|  | Glycan Biosynthesis and Metabolism | Lipopolysaccharide biosynthesis proteins | 198529 | 194963 | 14878 |
|  | Glycan Biosynthesis and Metabolism | N-Glycan biosynthesis | 19448 | 19943 | 1106 |
|  | Glycan Biosynthesis and Metabolism | Other glycan degradation | 174157 | 189721 | 14427 |
|  | Glycan Biosynthesis and Metabolism | Peptidoglycan biosynthesis | 367846 | 381880 | 24074 |
|  | Glycan Biosynthesis and Metabolism | Various types of N-glycan biosynthesis | 1816 | 2214 | 156 |
|  | Lipid Metabolism | alpha-Linolenic acid metabolism | 395 | 985 | 52 |
|  | Lipid Metabolism | Arachidonic acid metabolism | 16386 | 11903 | 1594 |
|  | Lipid Metabolism | Biosynthesis of unsaturated fatty acids | 44015 | 47318 | 2975 |
|  | Lipid Metabolism | Ether lipid metabolism | 100 | 111 | 11 |
|  | Lipid Metabolism | Fatty acid biosynthesis | 191969 | 209235 | 13636 |
|  | Lipid Metabolism | Fatty acid metabolism | 91146 | 103450 | 6619 |
|  | Lipid Metabolism | Glycerolipid metabolism | 131850 | 153546 | 11277 |
|  | Lipid Metabolism | Glycerophospholipid metabolism | 207632 | 239022 | 16420 |
|  | Lipid Metabolism | Linoleic acid metabolism | 25852 | 27247 | 1858 |
|  | Lipid Metabolism | Lipid biosynthesis proteins | 267594 | 279048 | 17466 |
|  | Lipid Metabolism | Primary bile acid biosynthesis | 9950 | 13685 | 1156 |
|  | Lipid Metabolism | Secondary bile acid biosynthesis | 9859 | 13476 | 1161 |
|  | Lipid Metabolism | Sphingolipid metabolism | 109089 | 121009 | 8821 |
|  | Lipid Metabolism | Steroid biosynthesis | 128 | 334 | 65 |
|  | Lipid Metabolism | Steroid hormone biosynthesis | 2212 | 2547 | 479 |
|  | Lipid Metabolism | Synthesis and degradation of ketone bodies | 10623 | 16048 | 1382 |
|  | Metabolism of Cofactors and Vitamins | Biotin metabolism | 71797 | 83862 | 5647 |
|  | Metabolism of Cofactors and Vitamins | Folate biosynthesis | 198209 | 212176 | 13510 |
|  | Metabolism of Cofactors and Vitamins | Lipoic acid metabolism | 21192 | 26437 | 2342 |
|  | Metabolism of Cofactors and Vitamins | Nicotinate and nicotinamide metabolism | 216505 | 221125 | 13918 |
|  | Metabolism of Cofactors and Vitamins | One carbon pool by folate | 319172 | 329497 | 21557 |
|  | Metabolism of Cofactors and Vitamins | Pantothenate and CoA biosynthesis | 301265 | 314558 | 19737 |
|  | Metabolism of Cofactors and Vitamins | Porphyrin and chlorophyll metabolism | 272896 | 293165 | 17422 |
|  | Metabolism of Cofactors and Vitamins | Retinol metabolism | 9446 | 10041 | 965 |
|  | Metabolism of Cofactors and Vitamins | Riboflavin metabolism | 109119 | 111597 | 7139 |
|  | Metabolism of Cofactors and Vitamins | Thiamine metabolism | 203254 | 230601 | 15207 |
|  | Metabolism of Cofactors and Vitamins | Ubiquinone and other terpenoid-quinone biosynthesis | 122727 | 120502 | 9077 |
|  | Metabolism of Cofactors and Vitamins | Vitamin B6 metabolism | 100790 | 100456 | 6695 |
|  | Metabolism of Other Amino Acids | beta-Alanine metabolism | 98151 | 107725 | 6906 |
|  | Metabolism of Other Amino Acids | Cyanoamino acid metabolism | 141283 | 137556 | 10265 |
|  | Metabolism of Other Amino Acids | D-Alanine metabolism | 45866 | 51294 | 3239 |
|  | Metabolism of Other Amino Acids | D-Arginine and D-ornithine metabolism | 333 | 123 | 28 |
|  | Metabolism of Other Amino Acids | D-Glutamine and D-glutamate metabolism | 75051 | 82971 | 5302 |
|  | Metabolism of Other Amino Acids | Glutathione metabolism | 78273 | 85759 | 5138 |
|  | Metabolism of Other Amino Acids | Phosphonate and phosphinate metabolism | 18977 | 22866 | 2150 |
|  | Metabolism of Other Amino Acids | Selenocompound metabolism | 138948 | 148378 | 9176 |
|  | Metabolism of Other Amino Acids | Taurine and hypotaurine metabolism | 53575 | 58022 | 3755 |
|  | Metabolism of Terpenoids and Polyketides | Biosynthesis of ansamycins | 33610 | 32850 | 2493 |
|  | Metabolism of Terpenoids and Polyketides | Biosynthesis of siderophore group nonribosomal peptides | 6875 | 5571 | 939 |
|  | Metabolism of Terpenoids and Polyketides | Biosynthesis of type II polyketide products | 97 | 356 | 56 |
|  | Metabolism of Terpenoids and Polyketides | Biosynthesis of vancomycin group antibiotics | 30279 | 34832 | 2282 |
|  | Metabolism of Terpenoids and Polyketides | Carotenoid biosynthesis | 2681 | 1919 | 237 |
|  | Metabolism of Terpenoids and Polyketides | Geraniol degradation | 27596 | 25315 | 1701 |
|  | Metabolism of Terpenoids and Polyketides | Limonene and pinene degradation | 46713 | 52549 | 3132 |
|  | Metabolism of Terpenoids and Polyketides | Polyketide sugar unit biosynthesis | 101114 | 109648 | 7084 |
|  | Metabolism of Terpenoids and Polyketides | Prenyltransferases | 166457 | 171965 | 10723 |
|  | Metabolism of Terpenoids and Polyketides | Terpenoid backbone biosynthesis | 271231 | 284800 | 17825 |
|  | Metabolism of Terpenoids and Polyketides | Tetracycline biosynthesis | 38429 | 49481 | 4635 |
|  | Metabolism of Terpenoids and Polyketides | Zeatin biosynthesis | 26584 | 24889 | 1896 |
|  | Nucleotide Metabolism | Purine metabolism | 961577 | 1039663 | 63835 |
|  | Nucleotide Metabolism | Pyrimidine metabolism | 858728 | 925153 | 57753 |
|  | Xenobiotics Biodegradation and Metabolism | 1,1,1-Trichloro-2,2-bis(4-chlorophenyl)ethane (DDT) degradation | 1 | 1 | 0 |
|  | Xenobiotics Biodegradation and Metabolism | Aminobenzoate degradation | 47506 | 57535 | 3503 |
|  | Xenobiotics Biodegradation and Metabolism | Atrazine degradation | 3935 | 5232 | 540 |
|  | Xenobiotics Biodegradation and Metabolism | Benzoate degradation | 75944 | 91486 | 6578 |
|  | Xenobiotics Biodegradation and Metabolism | Bisphenol degradation | 26730 | 30638 | 1999 |
|  | Xenobiotics Biodegradation and Metabolism | Caprolactam degradation | 6835 | 9448 | 674 |
|  | Xenobiotics Biodegradation and Metabolism | Chloroalkane and chloroalkene degradation | 59073 | 70451 | 5580 |
|  | Xenobiotics Biodegradation and Metabolism | Chlorocyclohexane and chlorobenzene degradation | 1573 | 3552 | 247 |
|  | Xenobiotics Biodegradation and Metabolism | Dioxin degradation | 10394 | 13576 | 1401 |
|  | Xenobiotics Biodegradation and Metabolism | Drug metabolism - cytochrome P450 | 6673 | 10940 | 896 |
|  | Xenobiotics Biodegradation and Metabolism | Drug metabolism - other enzymes | 151663 | 156755 | 10465 |
|  | Xenobiotics Biodegradation and Metabolism | Ethylbenzene degradation | 26623 | 27931 | 1992 |
|  | Xenobiotics Biodegradation and Metabolism | Fluorobenzoate degradation | 511 | 827 | 50 |
|  | Xenobiotics Biodegradation and Metabolism | Metabolism of xenobiotics by cytochrome P450 | 6614 | 10880 | 885 |
|  | Xenobiotics Biodegradation and Metabolism | Naphthalene degradation | 60570 | 63015 | 4204 |
|  | Xenobiotics Biodegradation and Metabolism | Nitrotoluene degradation | 23984 | 26114 | 2378 |
|  | Xenobiotics Biodegradation and Metabolism | Polycyclic aromatic hydrocarbon degradation | 42288 | 50286 | 3100 |
|  | Xenobiotics Biodegradation and Metabolism | Styrene degradation | 3421 | 5107 | 475 |
|  | Xenobiotics Biodegradation and Metabolism | Toluene degradation | 51043 | 54639 | 3285 |
|  | Xenobiotics Biodegradation and Metabolism | Xylene degradation | 9956 | 13132 | 1395 |
| Organismal Systems | Circulatory System | Cardiac muscle contraction | 550 | 378 | 108 |
|  | Digestive System | Bile secretion | 173 | 603 | 48 |
|  | Digestive System | Carbohydrate digestion and absorption | 7256 | 3817 | 797 |
|  | Digestive System | Mineral absorption | 1333 | 1113 | 122 |
|  | Digestive System | Protein digestion and absorption | 18781 | 14689 | 2007 |
|  | Endocrine System | Adipocytokine signaling pathway | 41202 | 37188 | 2883 |
|  | Endocrine System | GnRH signaling pathway | 13 | 1 | 0 |
|  | Endocrine System | Insulin signaling pathway | 27037 | 27277 | 1868 |
|  | Endocrine System | Melanogenesis | 1 | 1 | 1 |
|  | Endocrine System | PPAR signaling pathway | 51091 | 48723 | 3117 |
|  | Endocrine System | Progesterone-mediated oocyte maturation | 18985 | 19526 | 1296 |
|  | Endocrine System | Renin-angiotensin system | 51 | 47 | 2 |
|  | Environmental Adaptation | Circadian rhythm - plant | 18 | 19 | 5 |
|  | Environmental Adaptation | Plant-pathogen interaction | 56427 | 60768 | 4058 |
|  | Excretory System | Proximal tubule bicarbonate reclamation | 11966 | 15549 | 1377 |
|  | Excretory System | Vasopressin-regulated water reabsorption | 50 | 179 | 28 |
|  | Immune System | Antigen processing and presentation | 18985 | 19526 | 1296 |
|  | Immune System | Fc gamma R-mediated phagocytosis | 13 | 1 | 0 |
|  | Immune System | NOD-like receptor signaling pathway | 19363 | 19966 | 1330 |
|  | Immune System | RIG-I-like receptor signaling pathway | 365 | 349 | 43 |
|  | Nervous System | Glutamatergic synapse | 44480 | 48077 | 3010 |
| Unclassified | Cellular Processes and Signaling | Cell division | 40049 | 46659 | 3403 |
|  | Cellular Processes and Signaling | Cell motility and secretion | 76047 | 80180 | 4809 |
|  | Cellular Processes and Signaling | Electron transfer carriers | 3382 | 5688 | 487 |
|  | Cellular Processes and Signaling | Germination | 11105 | 9299 | 1290 |
|  | Cellular Processes and Signaling | Inorganic ion transport and metabolism | 73624 | 80081 | 5153 |
|  | Cellular Processes and Signaling | Membrane and intracellular structural molecules | 310415 | 308799 | 23649 |
|  | Cellular Processes and Signaling | Other ion-coupled transporters | 437694 | 476142 | 29823 |
|  | Cellular Processes and Signaling | Other transporters | 94586 | 98863 | 6206 |
|  | Cellular Processes and Signaling | Pores ion channels | 164400 | 168654 | 11672 |
|  | Cellular Processes and Signaling | Signal transduction mechanisms | 148024 | 162740 | 10696 |
|  | Cellular Processes and Signaling | Sporulation | 164562 | 170883 | 20586 |
|  | Genetic Information Processing | Protein folding and associated processing | 267958 | 286563 | 16755 |
|  | Genetic Information Processing | Replication, recombination and repair proteins | 273049 | 309434 | 21161 |
|  | Genetic Information Processing | Restriction enzyme | 85529 | 85017 | 5582 |
|  | Genetic Information Processing | Transcription related proteins | 1016 | 2402 | 239 |
|  | Genetic Information Processing | Translation proteins | 403971 | 438141 | 27506 |
|  | Metabolism | Amino acid metabolism | 97316 | 108614 | 6666 |
|  | Metabolism | Biosynthesis and biodegradation of secondary metabolites | 23457 | 22498 | 1796 |
|  | Metabolism | Carbohydrate metabolism | 55659 | 45136 | 3960 |
|  | Metabolism | Energy metabolism | 455614 | 486599 | 30882 |
|  | Metabolism | Glycan biosynthesis and metabolism | 18025 | 18893 | 1237 |
|  | Metabolism | Lipid metabolism | 49916 | 47009 | 3156 |
|  | Metabolism | Metabolism of cofactors and vitamins | 41687 | 46210 | 2569 |
|  | Metabolism | Nucleotide metabolism | 16524 | 21993 | 1736 |
|  | Metabolism | Others | 307966 | 341228 | 21875 |
|  | Poorly Characterized | Function unknown | 450385 | 510654 | 30993 |
|  | Poorly Characterized | General function prediction only | 1439931 | 1575793 | 97287 |
